# IMGT^®^ Nomenclature of Immunoglobulins (IG) or Antibodies and T Cell Receptors (TR): A Common Language for Immunoinformatics and Artificial Intelligence (AI)

**DOI:** 10.3390/antib15020035

**Published:** 2026-04-15

**Authors:** Marie-Paule Lefranc, Gérard Lefranc

**Affiliations:** 1 IMGT^®^, The International ImMunoGeneTics Information System^®^ (IMGT), Laboratoire d’ImmunoGénétique Moléculaire (LIGM), Institut de Génétique Humaine (IGH), Université de Montpellier (UM), Centre National de la Recherche Scientifique (CNRS), UMR 9002 CNRS-UM, 141 Rue de la Cardonille, CEDEX 5, 34396 Montpellier, France; 2 Institut Universitaire de France, 75231 Paris, France

**Keywords:** artificial intelligence (AI), IMGT-ONTOLOGY, ImMunoGeneTics (IMGT), immunoglobulin (IG) or antibody, immunoinformatics, T cell receptor (TR), copy number variation (CNV), haplotype, IMGT/mAb-DB, engineered variants of therapeutic antibodies

## Abstract

The immunoglobulins (IG) or antibodies and the T cell receptors (TR) are the antigen receptors of the adaptive immune responses (AIR) of jawed vertebrates (*Gnathostomata*). IMGT^®^, the international ImMunoGeneTics information system^®^, was created in 1989 by Marie-Paule Lefranc (Laboratoire d’ImmunoGénétique Moléculaire (LIGM), Université de Montpellier and CNRS) to deal with and to manage the huge diversity of IG or antibodies and TR. The founding of IMGT^®^ marked the advent of immunoinformatics, a new science which emerged at the interface between immunogenetics and bioinformatics. For the first time, the IG and TR variable (V), diversity (D), joining (J) and constant (C) genes were officially recognized as ‘genes’, as were the conventional genes. The IMGT-ONTOLOGY CLASSIFICATION axiom and the concepts of classification have generated the IMGT nomenclature and the IMGT Scientific chart rules for assigning IMGT names to IG and TR genes and alleles of *Homo sapiens* and of any other jawed vertebrate species. The IMGT nomenclature is used for genes in locus, in sequences (genomic or rearranged, expressed or not) and in structures enabling comparative immunology, evolutionary immunogenetics, standardized analysis and comparison of IG and TR repertoires analysis in normal or pathologic situations. IMGT nomenclature is used in basic, veterinary, and medical research, in clinical applications (mutation analysis in leukemia and lymphoma), and in therapeutic antibody design, engineering and humanization. By providing consistent and high standard biocuration for the description of the IG and TR loci, genes and alleles, and for the analysis of the IG or antibody and TR-expressed rearranged sequences and proteins and structures, the IMGT nomenclature is the common language for immunoinformatics and artificial intelligence (AI).

## 1. Introduction

IMGT^®^, the international ImMunoGeneTics information system^®^ (IMGT) [1] (https://www.imgt.org), was created in 1989 by Marie-Paule Lefranc (Laboratoire d’ImmunoGénétique Moléculaire (LIGM), Université de Montpellier (UM) and Centre National de la Recherche Scientifique (CNRS) at Montpellier, France. The objective was to deal with and to manage the huge diversity of the immunoglobulins (IG) or antibodies [2] and T cell receptors (TR) [3], which are the antigen receptors (AR) of the adaptive immune responses (AIR) [1,4,5,6]. For the first time, IG and TR variable (V), diversity (D), joining (J) and constant (C) genes [2,3,7,8] were officially recognized as ‘genes’, as were the conventional genes, and accepted in the Human Genome Mapping (HGM10) database. With this major breakthrough, the founding of IMGT^®^ marked the advent of immunoinformatics, a new science which emerged at the interface between immunogenetics and bioinformatics [1,9,10,11,12,13].

The specialist IG and TR nucleotide database, IMGT/LIGM-DB [14,15], was created by LIGM in collaboration with EMBL, using the same accession numbers as the GenBank, EMBL and DDBJ generalist databases (‘GEDI’) for data interoperability of the shared sequences. The first Internet connection of IMGT/LIGM-DB was organized for the 9th International Congress of Immunology (ICI) held in San Francisco, CA (USA) on 23–29 July 1995, marking the 7-year anniversary of the first Internet France–USA connection on the 28 July 1988. The creation of IMGT/LIGM-DB was followed by the development and implementation of other IMGT databases, tools and Web resources for the sequences, genes and structures of the IG or antibodies and TR, which together constitute the IMGT information system [1,9,10,11,12,13,14,15,16,17,18,19,20,21,22,23,24,25,26,27,28,29,30,31,32,33,34,35,36,37,38,39]. The IMGT data are identified, described, classified, numbered, localized, orientated and their source provided, based on the IMGT Scientific chart rules, themselves generated from the IMGT-ONTOLOGY axioms and concepts [1,10,12,25,40,41,42,43,44,45,46,47,48,49,50,51,52,53,54,55,56,57,58,59,60,61,62,63,64,65,66,67,68]. The IMGT standardization, initially characterized for the IG or antibodies and TR of jawed vertebrate species from fish to humans, has been extended to members of the immunoglobulin superfamily (IgSF) other than IG and TR, to the major histocompatibility (MH) proteins, to members of the MH superfamily (MhSF) other than MH and to related proteins of immune interest (RPI). Forty-five years of LIGM expert biocuration and innovation have made IMGT^®^, the international ImMunoGeneTics information system^®^, which comprises seven databases, seventeen tools and more than 25,000 pages of Web resources [1,34,35,36,37,38,39], the global reference for IG and TR data (genes, sequences and structures), with the IMGT nomenclature being the common language for immunogenetic and immunoinformatics.

Here, we firstly review, in three sections, the IMGT IG and TR key data which are the antigen receptors of the adaptive immune responses, the IMGT-ONTOLOGY and the IMGT information system which bridge biology and computational spheres, and the IMGT nomenclature (IMGT-NC) of the IG and TR genes and alleles, which marks the birth of immunoinformatics; we then present the IMGT-NC reports for novel IG and TR genes and alleles names, and, in three sections, the IMGT-NC new concepts for biocuration of IG and TR loci from genome assemblies, for gene copy number variations (CNV) with the definition of ‘CNV haplotypes’ and ‘CNV-Locus-Haplotype’, and for engineered IGHG variants for effector properties, half-life and structures of therapeutic antibodies. The last section highlights examples of long term biological contribution of researchers using the IMGT nomenclature, for the genomic and expressed repertoires of the IG and TR of jawed vertebrates, from fish to humans, for antibody engineering, humanization, structure and specificity, demonstrating that IMGT-NC is a common long-term scientific research endeavor, which has provided a foundational and unifying infrastructure for immunogenetics in immunoinformatics, which is now available for artificial intelligence.

## 2. IMGT Key Data: The IG and TR, Antigen Receptors of the Adaptive Immune Responses

**Figure 1:**
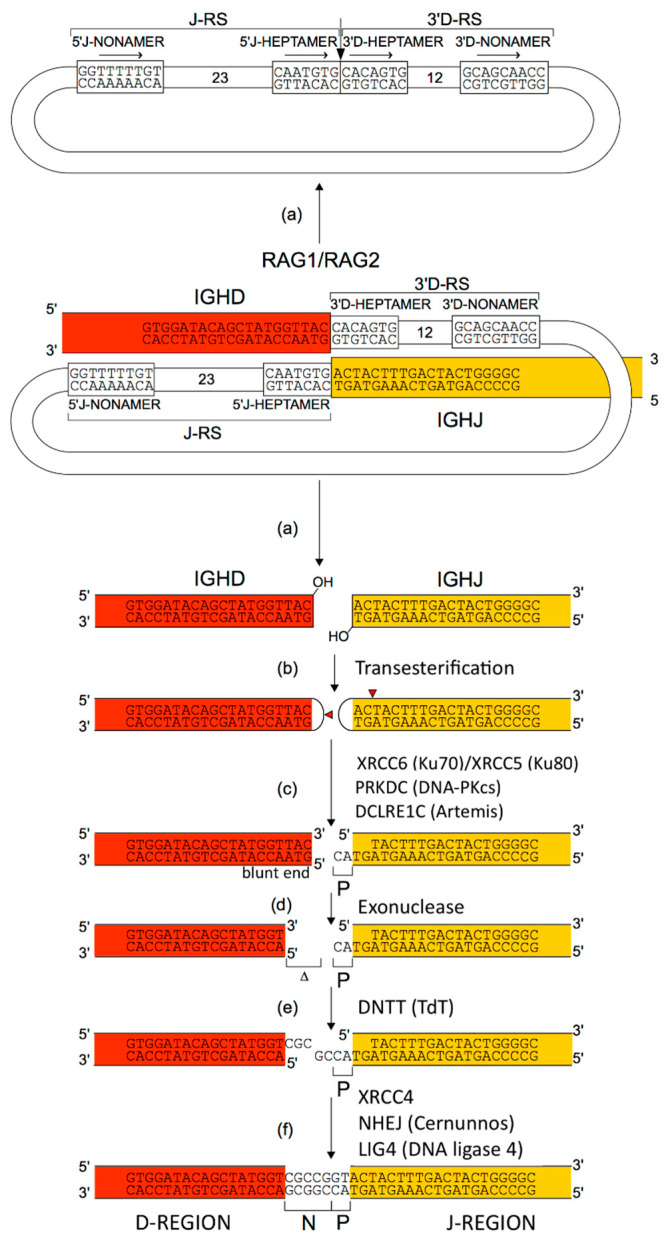
Molecular events generating the junction N-diversity, in a rearrangement between a D gene and a J gene in the IGH locus [6]. (**a**) The enzymatic RAG1/RAG2 protein complex recognizes the recombination signal (RS) sites and cuts the germline DNA between the heptamer and the coding region. The signal joint sequence (upward arrow) is eliminated as an excision circle, whereas at each coding end, here 3′D and 5′J ends (downward arrow), (**b**) a hairpin is formed by transesterification. (**c**) The hairpin is cut by the protein complex (X-ray repair cross complementing 6 (XRCC6, Ku70)/X-ray repair cross complementing 5 (XRCC5, Ku80), protein kinase DNA-activated catalytic subunit (PRKDC, DNA-PKcs, DNA-PK), DNA cross-link repair 1C (DCLRE1C, Artemis)). Depending on the position of the cut-off site, a blunt end or P nucleotides are obtained. (**d**) An exonuclease eliminates nucleotides at the coding ends. (**e**) DNA nucleotidylexotransferase (DNTT, TdT, terminal deoxynucleotidyl transferase) adds N nucleotides, preferably ‘g’ and ‘c’ at random without template. (**f**) Finally, the rearranged DNA is repaired and ends are ligated by the ligase complex (X-ray repair cross complementing 4 (XRCC4), non-homologous end joining factor 1 (NHEJ1, Cernunnos), LIG4 (DNA ligase 4, ligase IV DNA ATP-dependent). D-REGION IGHD (red), J-REGION IGHJ (yellow). (With permission from M-P. Lefranc and G. Lefranc, LIGM, Founders and Authors of IMGT^®^, the international ImMunoGeneTics information system^®^, http://www.imgt.org).

The adaptive immune response (AIR) was acquired by jawed vertebrates (or *Gnathostomata*) more than 450 million years ago and is found in all extant jawed vertebrate species from fish to humans [1]. The adaptive immune response is characterized by a remarkable immune specificity and memory, which are properties of the B and T cells owing to an extreme diversity of their specific antigen receptors, the IG or antibodies of the B cells (membrane IG) and of the plasmocytes (secreted IG) [2,4,5,6] and the TR [3]. The potential expressed antigen receptor repertoire (ARR) of the adaptive immune responses (AIR) of each individual is estimated to comprise about 2 × 10^12^ different IG and TR, and the limiting factor is only the number of B and T cells that an organism is genetically programmed to produce [1]. This huge diversity results from the complex molecular synthesis of the IG and TR chains, and more particularly of the synthesis of the variable domain (V-DOMAIN) that, at the N-terminal end of each chain, recognizes and binds the antigens [1,2,3,4,5,6]. The IG and TR synthesis includes several unique mechanisms that occur at the DNA level: (1) combinatorial rearrangements of the V, D and J genes that code the V-DOMAIN (the number of V, D and J genes in a given IG or TR locus represents the potential genomic repertoire), (2) exonuclease trimming at the ends of the V, D and J genes and random addition of nucleotides by the DNA nucleotidylexotransferase (DNTT, TdT, terminal deoxynucleotidyl transferase) that creates the junctional N-diversity regions (Figure 1) [6] before ligation of the V-(D)-J gene (the V-(D)-J-REGION thus created is then classically transcribed into mRNA and spliced, in the chain transcript, to the C-REGION exon(s) encoded by the C-gene), and (3) later during B cell differentiation, for the IG, two unique mechanisms again at the DNA level, somatic hypermutations (SHM) and class or subclass switch recombination (CSR) [6].

**Figure 2:**
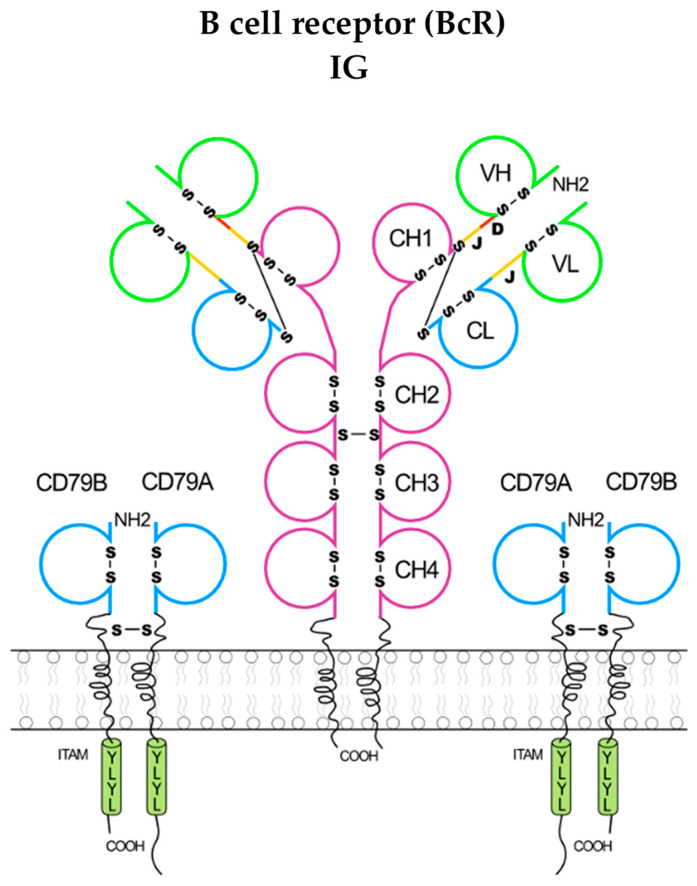
B cell receptor (BcR) [2,6]. The B cell receptor (BcR) on the surface of mature B cells comprises an immunoglobulin (IG) or antibody, here IgM, as a monomer H2L2, anchored in the membrane of the B cell (membrane IG or mIG) and the CD79 signaling coreceptors constituted of two heterodimers CD79A/CD79B (BcR = mIG + dimeric CD79A/CD79B coreceptors). VH (green), CH1, CH2, CH3 and CH4 (pink) indicate the domains of the H-mu chains of the IgM. Depending on the light chain type, L-kappa or L-lambda, VL (green) and CL (blue) correspond to V-kappa and C-kappa, or to V-lambda and C-lambda, respectively. The CD79A and CD79B belong to the IgSF by their extracellular C-like domains (blue). ITAM motifs (green cylinders, not to scale) are indicated schematically by the letters YLYL with Y (tyrosyl) and L (leucyl or isoleucyl) amino acids. ITAM are rich in tyrosines and with a consensus (D/E)xxYxx(L/I)x6–8Yxx(L/I) [6]. Cross-linking of the BcR induces the tyrosylphosphorylation of the ITAM on the cytoplasmic region of CD79A and CD79B, and the signaling cascade leading to B cell activation, by recruitment of signaling molecules which belong to families of protein tyrosine kinases (PTKs), the Src family (LYN, BLK, FYN), the Syk family (SYK) and the Tec family (BTK), and provide signal transmission (with permission from M-P. Lefranc and G. Lefranc, LIGM, Founders and Authors of IMGT^®^, the international ImMunoGeneTics information system^®^, https://www.imgt.org).

**Figure 3:**
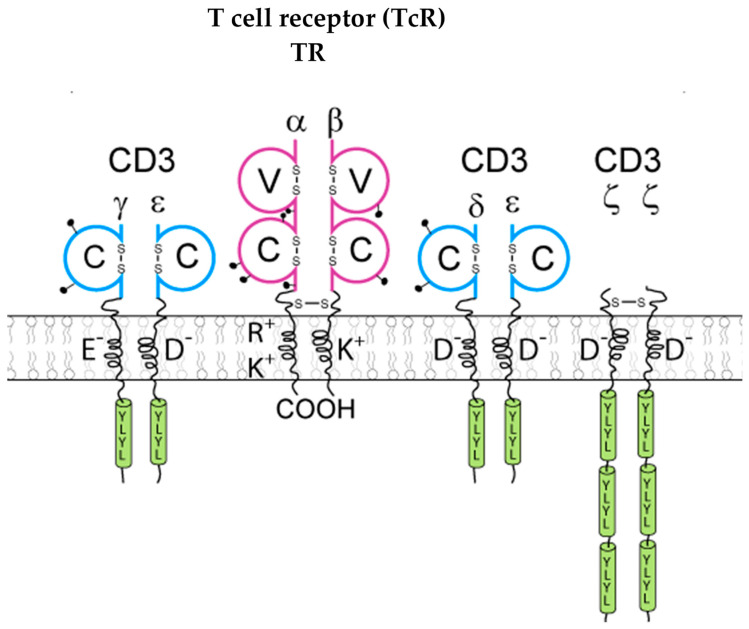
T cell receptor (TcR) [3]. The T cell receptor (TcR) on the surface of T cells comprises a heterodimeric T cell receptor (TR) alpha–beta (or gamma–delta), anchored in the membrane of the T cells and the CD3 signaling coreceptors constituted of two heterodimers CD3G/CD3E and CD3D/CD3E and of the homodimer CD247 (alias CD3Z) [1,3]. Depending on the TR chain type, alpha and beta, or gamma and delta, the variable (V) domains (pink) are V-alpha and V-beta, or V-gamma and V-delta, and the constant (C) domains (pink) are C-alpha and C-beta, or C-gamma and C-delta, respectively. The CD3G, CD3D, CD3E belong to the IgSF due to their extracellular C-like domains (blue). The N-glycosylation sites are those of a TR alpha–beta. Positively charged (lysyl K^+^, arginyl R^+^) and negatively charged (glutamyl E^−^, aspartyl D^−^) amino acids of the transmembrane region are shown. ITAM motifs (green cylinders, not to scale) are indicated schematically by the letters YLYL for Y (tyrosyl) and L (leucyl or isoleucyl) amino acids. (With permission from M-P. Lefranc and G. Lefranc, LIGM, Founders and Authors of IMGT^®^, the international ImMunoGeneTics information system^®^, https://www.imgt.org).

The immunoglobulins (IG) [1,2,4,5,6] or antibodies and the T cell receptors (TR) [1,3] are the antigen receptors of the adaptive immune responses (AIR) [1]. The IG are expressed as B cell receptors (BcR) at the surface of B cells with the coreceptors CD79A/CD79B on mature and memory B cells (Figure 2) whereas the TR are expressed as T cell receptors (TcR) at the surface of T cells with the coreceptors CD3G/CD3E, CD3D/CD3E, and CD247 (alias CD3Z)/CD247 (Figure 3).

The coreceptors of the BcR and TcR transduce the signal intracellularly following the binding of the antigen receptor, IG and TR, respectively, to its antigen [2,3]. The IG recognize antigens in their native (unprocessed) form, whereas the TR recognize processed antigens that are presented as peptides by the highly polymorphic major histocompatibility (MH, in humans HLA for human leucocyte antigens) proteins [1]. At the terminal stage of the B cell differentiation, the IG are secreted by the plasmocytes (or plasma cells, or effector B cells) found in the bone marrow, lymph nodes and spleen [2,6], whereas the TR remain always membranar on the T cells [3].

## 3. IMGT: An Ontology and a System to Bridge Biology and Computational Spheres

### 3.1. IMGT-ONTOLOGY: Accuracy and Consistency of the IMGT Data

The accuracy and the consistency of the IMGT data (genes, nucleotide and amino acid sequences and structures), as well as the coherence between the different IMGT components (databases, tools and Web resources) are based on IMGT-ONTOLOGY, the first ontology for immunogenetics and immunoinformatics [1,10,12,25,40,41,42,43,44,45,46,47,48,49,50,51,52,53,54,55,56,57,58,59,60,61,62,63,64,65,66,67,68]. The IMGT-ONTOLOGY comprise seven axioms ‘IDENTIFICATION’ [50], ‘DESCRIPTION’ [51], ‘CLASSIFICATION’ [52], ‘NUMEROTATION’ [53,54,55,56,57,58,59,60,61,62,63,64,65,66], ‘LOCALIZATION’ [48], ‘ORIENTATION’ [48,49] and ‘OBTENTION’ [48,49], that postulate that objects, processes and relations have to be identified, described, classified, numerotated, localized, and orientated, and that the way they are obtained has to be determined [40,43,44,47,48,49].

**Figure 4:**
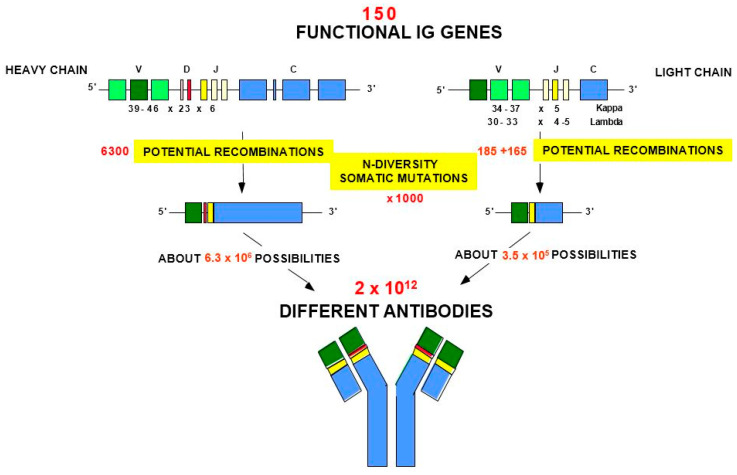
Molecular synthesis of the immunoglobulins (IG) or antibodies and origin of their diversity [2,6]. The gene numbers are from the human (*Homo sapiens*) IGH, IGK and IGL loci [5]. The molecular mechanisms creating the diversity of the antigen receptors of the adaptive immune responses include the combinatorial diversity (V-(D)-J rearrangements), the junctional diversity (including the N-diversity), the somatic hypermutations (SHM) and the pairing of the heavy and light chains. Altogether the mechanisms of diversity that occur at the DNA level in the B cell result in about 6.3 × 10^6^ and about 3.5 × 10^6^ possibilities of heavy and light chains, respectively, and the pairing of one heavy chain with one light chain (the antibody is made of two identical heavy and light chains) results in a potential repertoire of 2 × 10^12^ different antibodies. The chain pairing results from two inter-H-L and from inter-H-H disulfide bridges which depend on IG or antibody class or subclass. Only the core gene regions are represented. V = V-REGION (in green), D = D-REGION, J = J-REGION, C = C-REGION (in blue). The gene regions involved in the H chain V-D-J and L chain V-J rearrangements are highlighted: V (in dark green), D (in red) and J (in yellow). (With permission from M-P. Lefranc and G. Lefranc, LIGM, Founders and Authors of IMGT^®^, the international ImMunoGeneTics information system^®^, https://www.imgt.org).

**Figure 5:**
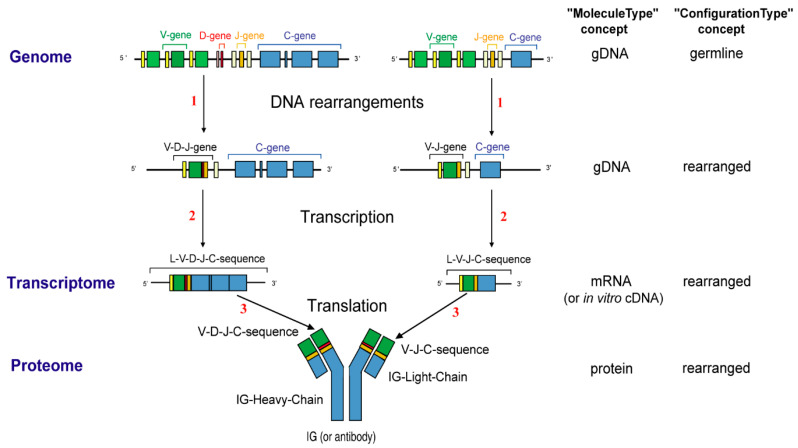
An example of biological knowledge at the molecular level: the synthesis of an IG or antibody in humans [2,6]. Compared with Figure 3, molecular entity types of the IG synthesis (IDENTIFICATION axiom) [50] in jawed vertebrates (*Gnathostomata*) are indicated as keywords. (1) DNA rearrangements (is_rearranged_into). (2) Transcription (is_transcribed_into). (3) Translation (is_translated_into). IMGT standardized keywords, generated from the concepts of identification, identify the entity types based on the MoleculeType and the ConfigurationType. The MoleculeType is gDNA, mRNA (or in vitro cDNA) or protein. The ConfigurationType is undefined (conventional gene or C-gene), germline (V-gene, D-gene and J-gene) or rearranged. The rearranged entities include V-D-J-gene (label L-V-D-J-GENE) and V-J-gene (label L-V-J-GENE) (gDNA), L-V-D-J-C-sequence and L-V-J-C-sequence (cDNA), V-D-J-C-chain and V-J-C-chain (protein) [50]. The functionality of undefined and germline entities is functional (F), open reading frame (ORF) or pseudogene (P) [50]. The functionality of rearranged entities is productive or unproductive [50]. L = L-REGION (shown in ‘Transcriptome’) (light yellow), V = V-REGION (in green), D = D-REGION, J = J-REGION, C = C-REGION (in blue). The gene core regions involved in the V-D-J (heavy chain) and V-J (light chain) rearrangements are highlighted: V (in dark green), D (in red) and J (in orange yellow). (With permission from M-P. Lefranc and G. Lefranc, LIGM, Founders and Authors of IMGT^®^, the international ImMunoGeneTics information system^®^, https://www.imgt.org).

The IMGT-ONTOLOGY seven axioms and generated concepts have been essential for the conceptualization of molecular immunogenetics knowledge through diverse facets (Formal IMGT-ONTOLOGY or IMGT-Kaleidoscope [47]) and for the definition of the IMGT Scientific chart rules (https://www.imgt.org/IMGTScientificChart/) (accessed on 11 February 2026) (IMGT-Choreography) [43,44]. The molecular synthesis of immunoglobulins (IG) or antibodies and the origin of their diversity (Figure 4) is shown, as an example of biological knowledge at the molecular level, based on the IMGT-ONTOLOGY concepts of identification [50] (Figure 5).

Detailed molecular information on the synthesis of the H-mu chains (following D-J and V-D-J rearrangements in the IGH locus), the synthesis of the L-kappa and L-lambda chains (following V-J rearrangements in the IGK and IGL loci), the origin of the variable domain diversity of the immunoglobulins, the class or subclass switch recombination, the regulation of the rearrangements and chain expression, the structural and biological properties of the secreted immunoglobulins, the human IG classes and subclasses, heavy and light chain types, and the *Homo sapiens* immunoglobulin (IG) chain characteristics taking into account the IMGT concepts have been reviewed elsewhere [6].

The IDENTIFICATION axiom and concepts of identification led to the standardized keywords and qualifiers and to their definitions (e.g., reference sequence (https://www.imgt.org/IMGTScientificChart/SequenceDescription/IMGTreferencesequences.html (accessed on 9 April 2026), clonotype, paratope, epitope, allotype, variant, Fc receptor, FcR) and to the relations for molecule identification [50]) (IMGT standardized keywords: https://www.imgt.org/IMGTScientificChart/SequenceDescription/Keywords.php (accessed on 9 April 2026); IMGT/LIGM-DB qualifiers: https://www.imgt.org/ligmdb/qualifier (accessed on 9 April 2026)).

The DESCRIPTION axiom and concepts of description led to the standardized labels (e.g., V-REGION, complementarity determining region (CDR)-IMGT (CDR1-IMGT to CDR3-IMGT) and framework region (FR-IMGT) (FR1-IMGT to FR4-IMGT)) and to the relations for the prototype descriptions [51] (IMGT/LIGM-DB standardized labels: https://www.imgt.org/ligmdb/label#) (accessed 14 February 2026). IMGT standardized keywords and labels have first been defined for nucleotide sequences and their translation (Supplementary Materials Table S1. List of definitions in the Encyclopedia of Systems Biology). For sequences analyzed only at the amino acid level (e.g., from crystallized three-dimensional (3D) structures), the identification (IMGT keywords) and description (IMGT labels) are done in terms of domains, chains and receptors (IMGT/PROTEIN-DB and IMGT/3Dstructure-DB: Standardized keywords and labels for IG, TR, MH, RPI and FPIA: https://www.imgt.org/IMGTScientificChart/SequenceDescription/IMGT3Dkeywords.html (accessed on 9 April 2026)).

**Figure 6:**
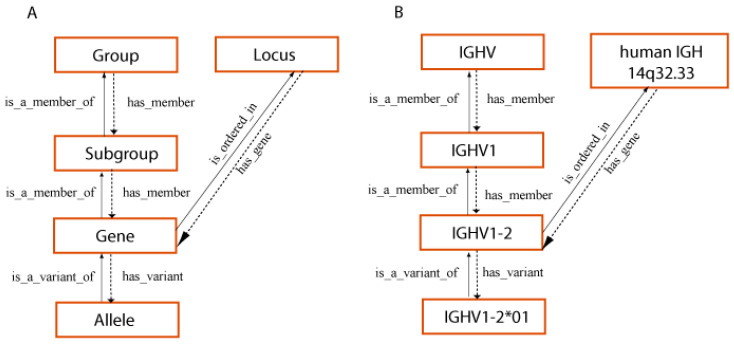
Concepts of classification for IMGT gene and allele nomenclature (CLASSIFICATION axiom) [52,67,68]. (**A**) Hierarchy of the concepts of classification and their relations. The definition of the reciprocal relations between concepts can be read, from one concept to the other, either ascending the hierarchy (solid arrows) or descending the hierarchy (dotted arrows). (**B**) Examples of concept instances for each concept of classification. The concept instances are associated with an instance of the “Taxon” concept (IDENTIFICATION axiom), and more precisely for the “Gene” and “Allele” concepts to an instance of the “Species” concept (here, *Homo sapiens*) [1,2,3,6,7,8]. The “Locus” concept is a concept of localization (LOCALIZATION axiom) [48]. It is shown with the reciprocal relations to the “Gene” concept. (With permission from M-P. Lefranc and G. Lefranc, LIGM, Founders and Authors of IMGT^®^, the international ImMunoGeneTics information system^®^, https://www.imgt.org).

The CLASSIFICATION axiom and concepts of classification [1,52] have formalized the rules of the IMGT standardized gene and allele nomenclature of the IG and TR of jawed vertebrates (*Gnathostomata*), conceived by LIGM [1,2,3,7,8,52,66,67,68], starting from the early eighties as the ImMunoGeneTics nomenclature (IMGT-NC), officially recognized in 1989 at the 10th Human Genome Mapping (HGM10) Workshop, and demonstrated by the publication of ‘The Immunoglobulin FactsBook’ [2] and ‘The T cell receptor FactsBook’ [3] in 2001 and the creation of the WHO-IUIS Nomenclature Subcommittee for Immunoglobulins and T cell receptors (IMGT-NC) in 2007 [67,68]. The CLASSIFICATION axiom and concepts of classification [1,52] (Figure 6) provide identical IMGT Scientific chart rules regarding the gene and allele nomenclature across jawed vertebrates from fish to humans [1,2,3,7,8,52,66,67,68] and is one of the two pillars of immunoinformatics [66].

The NUMEROTATION axiom and concepts of numerotation [1,53,54,55,56,57,58,59,60,61,62,63,64,65,66] are at the origin of the IMGT unique numbering for the variable (V) domains of the IG and TR [55], of the IMGT unique numbering for the constant (C) domains of the IG and TR [56] and of the IMGT unique numbering of the groove (G) domains of the MH [57], with their graphical representations, the IMGT Colliers de Perles for V, C and G domains [61,62,63,64,65,66]. The IMGT unique numbering for the V and C domains of the IG and TR is valid, respectively, for the V-LIKE and C-LIKE domains of the IgSF members other than IG and TR from vertebrate and invertebrate species [55,56,58,60]. Similarly, the IMGT unique numbering for the G domains of the MH is valid for the G-LIKE domains of the MhSF members other than MH [57,58,60]. The NUMEROTATION axiom and concepts of numerotation [1,53,54,55,56,57,58,59,60,61,62,63,64,65,66], which provide an IMGT unique numbering for V, C and G domains whatever the species, represent the second pillar of immunoinformatics [66] (Table 1). IMGT positions per domain are used in sections of the IMGT Repertoire IG and TR (protein displays, alignments of alleles, CDR-IMGT lengths, allotypes), and to number amino acids involved in paratope/epitope (antigen receptor IG or TR V-domain/target interactions) and in effector properties (antigen receptor IG C-domain/effector binding proteins (e.g., FcγR, complement interactions).

### 3.2. Relations Between the Concepts of IMGT-ONTOLOGY Implemented in the IMGT Information System

#### 3.2.1. Overall View of the Relations Between Concepts at the Molecular Level

**Figure 7:**
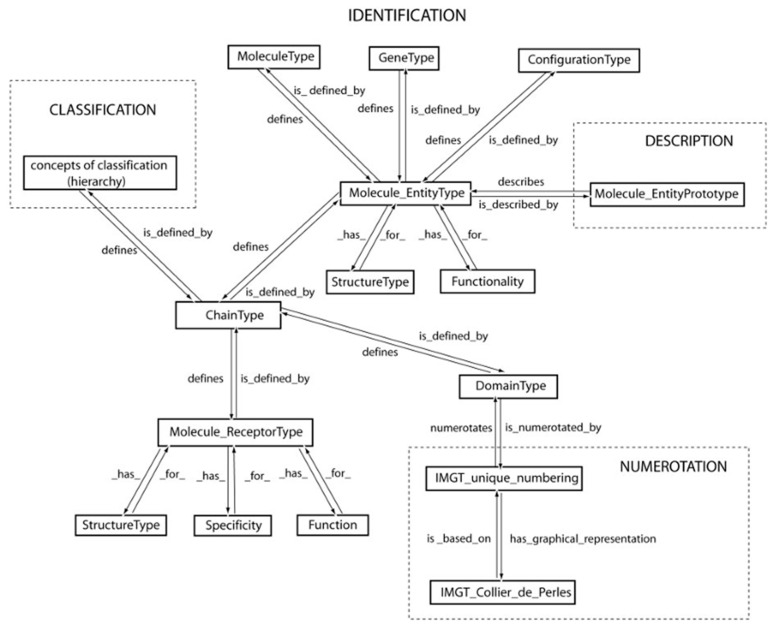
Relations between the concepts of IMGT-ONTOLOGY implemented in the IMGT system. Main IMGT-ONTOLOGY concepts of identification [50] generated from the IDENTIFICATION axiom [50] and their relations with concepts generated from the DESCRIPTION [51], CLASSIFICATION [52] and NUMEROTATION [55,56,57,58,64,65] axioms, at the molecular level. (With permission from M-P. Lefranc and G. Lefranc, LIGM, Founders and Authors of IMGT^®^, the international ImMunoGeneTics information system^®^, https://www.imgt.org).

The relations between the concepts of identification, description, classification and numerotation implemented in the databases, tools and web resources of the IMGT^®^ information system [13,14,15,16,17,18,19,20,21,22,23,24,25,26,27,28,29,30,31,32,33,34,35,36,37,38,39] and formalized in IMGT-ONTOLOGY [12,40,41,42,43,44,45,46,47,48,49,50,51,52,53,54,55,56,57,58,59,60,61,62,63,64,65,66,67,68] are summarized in Figure 7.

#### 3.2.2. The ‘Molecule_EntityType’ Concept

The ‘Molecule_EntityType’ concept (Figure 7) allows us to identify any coding molecule of any jawed vertebrate genome, transcriptome and proteome. There are 21 entities defined by: ‘MoleculeType’ (‘gDNA’, ‘mRNA’ (or in vitro ‘cDNA’), ‘protein’), ‘GeneType’ [‘conventional’, and for the IG and TR, ‘variable’ (V), ‘diversity’ (D), ‘joining’ (J) and ‘constant’(C)], and ‘ConfigurationType’ (‘undefined’ for conventional and C genes, ‘germline’ for unrearranged V, D and J genes and ‘rearranged’ for V, D and J genes after DNA rearrangements [1,3]). Three entities, ‘gene’, ‘nt-sequence’ and ‘AA-sequence’, respectively, identify the gDNA, mRNA (or in vitro cDNA) and protein (‘MoleculeType’) of a conventional gene (‘GeneType’), which by definition is an undefined configuration (‘ConfigurationType’). Eighteen entities identify the IG and TR. The ten most classical IG and TR entity types comprise V-gene, D-gene, J-gene, C-gene, V-D-J-gene, V-J-gene, L-V-D-J-C-sequence, L-V-J-C-sequence, V-D-J-C-sequence and V-J-C-sequence (Table 2).

For example, the entity ‘V-gene’ identifies a ‘gDNA’ containing a ‘V’ gene, in ‘germline’ configuration. The entity ‘L-V-J-C-sequence’ identifies a sequence of ‘mRNA’ (or in vitro ‘cDNA’) containing ‘V’, ‘J’ and ‘C’ genes, with V and J in ‘rearranged’ configuration. The less classical eight entities correspond to partial rearrangements or to sterile transcripts [47].

A ‘Molecule_EntityType’ concept entity has two properties, defined by ‘StructureType’ and ‘Functionality’ (Figure 7). The ‘StructureType’ concept allows us to identify instances with a classical organization (‘regular’), from those that have been modified either naturally in vivo (‘orphon’, ‘processed orphon’, ‘unprocessed orphon’, ‘unspliced’, ‘partially spliced’, etc.) or artificially in vitro (‘chimeric’, ‘humanized’, ‘transgene’, etc.). The ‘Functionality’ concept includes five entities: three of them, ‘functional’, ‘ORF’ (open reading frame) and ‘pseudogene’ identify the functionality of ‘Molecule_EntityType’ instances in undefined or germline configuration (conventional genes, C genes, germline V, D and J genes), whereas the two others, ‘productive’ and ‘unproductive’, identify the functionality of ‘Molecule_EntityType’ instances in rearranged configurations (rearranged V, D and J genes, fusion genes resulting from translocations or obtained by biotechnology and/or molecular engineering).

#### 3.2.3. The ‘ChainType’ Concept

**Figure 8:**
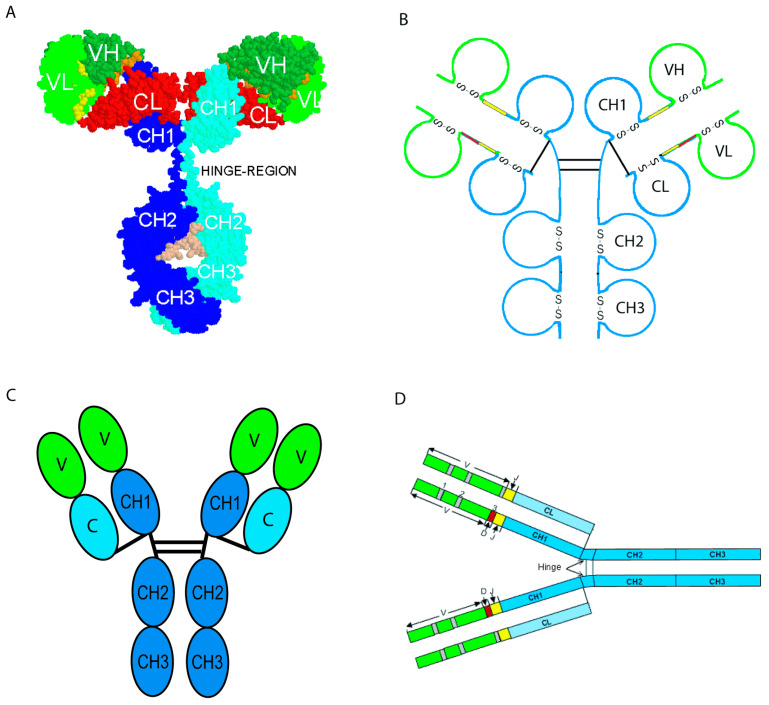
Identification of an IG or antibody as an entity of the ‘Molecule_ReceptorType’ made of four chains, two IG-Heavy-Chain and two IG-Light-Chain (‘ChainType’concept) (IDENTIFICATION) [50]. The four representations, although different, allow us to identify an IG as a receptor of four chains, themselves organized in domains (‘DomainType’ concept). VH and VL are V type domains, coded by the V-D-J-region and V-J-region, respectively. CL, CH1, CH2 and CH3 are C type domains. The C type domain codes the constant region (CL for the IG-Light-Chain and CH1, hinge, CH2 and CH3 for the IG-Heavy-Chain. (**A**) IG or antibody 3D molecular model with labeled VH, CH1, HINGE-REGION, CH2, CH3 for the heavy (H) chains and VL, CL for the light (L) chains, (**B**) IG or antibody organization in twelve IG labeled domains (green border for the four V domains and blue border for the eight C domains), with the conserved intra-domain disulfide bridge (S-S) for each. Each H-chain is connected to an L-chain by a H-L bridge. The two H-chains are connected by H-H disulfide bridges (here, two) at the hinge level, (**C**) IMGT IG or antibody representation with each of the twelve domains shown as an ovoid module (green for the VH and pale green for the VL, blue for the CH1, CH2 and CH3 and pale blue for the CL), used to illustrate the different formats of therapeutic antibodies (e.g., in IMGT/mAb-DB), (**D**) IG or antibody with a linear representation of the regions coded by the V, D, J and C gene types. The V, D, J regions which encode the variable domains are highlighted: VH is encoded by a rearranged V-D-J-region, and VL by a rearranged V-J-region, where V is in green, D is in red and J in yellow. This representation, schematized as a Y shape, is frequently used to represent an IG or antibody. (With permission from M-P. Lefranc and G. Lefranc, LIGM, Founders and Authors of IMGT^®^, the international ImMunoGeneTics information system^®^, https://www.imgt.org).

The ‘ChainType’ concept (Figure 7) identifies the type of chain. It is one of the most important concepts of identification for the standardization of genome, transcriptome and proteome data in system biology. Indeed, an instance of the ‘ChainType’ concept is defined not only by an entity of the ‘Molecule_EntityType’ (V-J-C-sequence or V-D-J-C-sequence) but also by an entity of a concept of classification and by a definition in domains, which bridges the gap with 3D structures. Moreover, it is the chain composition which defines the ‘Molecule_ReceptorType’ concept and identifies the type of protein receptor [47]. Thus, IG is an entity of the ‘Molecule_ReceptorType’ concept, defined as comprising four chains, two IG-Heavy and two IG-Light chains (entities of the ‘ChainType’ concept), which are identical two by two and covalently linked. Four graphical representations of an IG or antibody are classically used (Figure 8): (A) 3D molecular model, (B) organization in 12 labeled domains with the conserved intra-domain disulfide bond (S-S) shown for each, (C) representation with each of the 12 domains as an ovoid module (green for the VH and pale green for the VL, blue for the CH1-CH3 and pale blue for the CL (e.g., in IMGT/mAb-DB)), and (D) linear representations of the regions coded by the V (in green), D (in red), J (in yellow) and C (in blue) gene types [66].

By its relation with the concepts of classification, the ‘ChainType’ concept contains a hierarchy of concepts that identify the chain type at different levels of granularity. The finest level of granularity, the ‘GeneLevelChainType’ concept, identifies the chain type by reference to the gene(s), which code(s) the chain (reciprocal relations ‘is_coded_by’ and ‘codes’). The number of instances of the ‘GeneLevelChainType’ concept depends on the number of functional genes and ORF per haploid genome in a given species (in the case of the IG and TR, it is the number of functional and ORF constant genes, which is taken into account). If only the functional genes are considered, the instances of this concept correspond to the isotypes.

#### 3.2.4. The ‘DomainType’ Concept

A chain type instance can also be defined by its constitutive structural units (‘DomainType’concept). A domain is a chain subunit characterized by its 3D structure and, by extension, its amino acid sequence and the nucleotide sequence that encodes it. The ‘DomainType’ concept may theoretically comprise many instances, but so far only the instances that have been carefully characterized by LIGM have been entered in IMGT-ONTOLOGY. The ‘DomainType’concept currently has three major instances fully characterized: V type domain (variable domains of the IG and TR and V-like domains of IgSF superfamily members other than IG or TR) [55], C type domain (constant domains of the IG and TR and C-like domains of IgSF superfamily members other than IG or TR) [56] and G type domain (groove domains of the major histocompatibility (MH) proteins and G-like domains of MhSF superfamily members other than MH) [57] (https://www.imgt.org/IMGTindex/Domain.php) (accessed on 14 February 2026). Other instances of ‘DomainType’ from RPI in IMGT/DomainDisplay [1] include representatives of the A type domain (e.g., [D1] to [D4] of F11 (coagulation factor XI, P03951) and of KLKB1 (kallikrein B1, P039952)), the F type domain (108 sequences of F domains of FN1 (fibronectin 1) and DSCAM (DS cell adhesion molecule)) and the S domain of the scavenger receptor superfamily (SrSF) (e.g., S domains of CD5, CD6, CD163, CD163L1).

#### 3.2.5. The ‘MoleculeReceptorType’ Concept

The properties of the ‘Molecule_ReceptorType’ concept include structure, specificity and function (Figure 6). Thus, instances of the ‘Specificity’ concept identify the antigen recognized by an antigen receptor (IG or TR). The instances of that concept (several hundreds at the present time) can be connected on the one hand, with the ‘Epitope’ concept that identifies the part of the antigen recognized by the antigen receptor and, on the other hand, with the ‘Paratope’ concept that identifies the part of the antigen receptor (IG or TR), which recognizes and binds to the antigen [1]. Instances of the ‘Function’ concept identify the dual function of the IG or antibodies [5] and the modifications linked to the allotypes [66] and to the IMGT engineered variants [66].

#### 3.2.6. The ‘Molecule_EntityPrototype’ Concept (DESCRIPTION)

**Figure 9:**
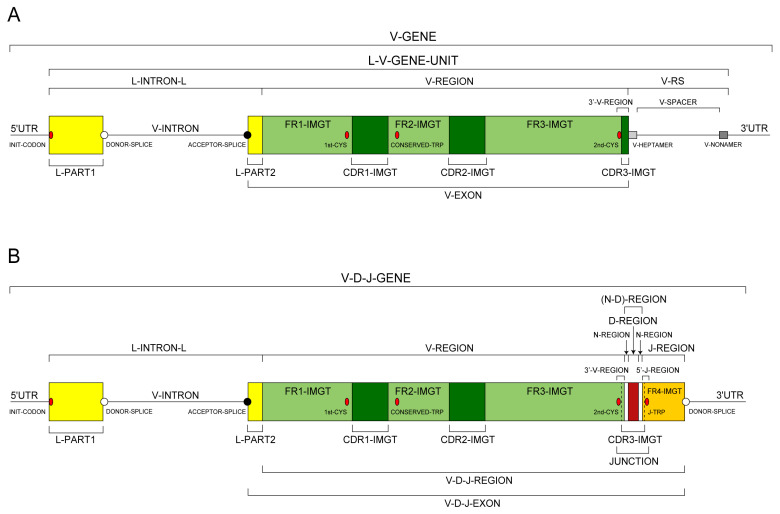
IMGT^®^ standardized labels of the “V-GENE” and “V-D-J-GENE” “Molecule_ EntityPrototype” (DESCRIPTION). (**A**) “V-GENE” prototype. The genomic (gDNA) molecular organization of a V-GENE (germline configuration) highlights the 5′UTR, L-PART1 (yellow), V-INTRON, V-EXON (made of the L-PART2 (yellow) and V-REGION (green)) and 3′UTR (starting with the V-RS). The V-REGION includes FR1-IMGT (with 1st-CYS), CDR1-IMGT, FR2-IMGT (with CONSERVED-TRP), CDR2-IMGT, FR3-IMGT (with 2nd-CYS) and germline CDR3-IMGT. (**B**) “V-D-J-GENE” prototype. The genomic (gDNA) molecular organization of a V-D-J-GENE (rearranged configuration) features a V-D-J-REGION which encodes a VH domain. Owing to the V-D-J rearrangement, the V-REGION is joined to an additional (N-D)-REGION (D-REGION (red) between N-REGION (white)) and J-REGION (pale orange). The CDR3-IMGT extends between 2nd-CYS and J-TRP (which are the CDR3-IMGT anchors and belong to the FR3-IMGT and FR4-IMGT, respectively). The JUNCTION includes the two anchors 2nd-CYS and J-TRP and therefore is two amino acids longer than the CDR3-IMGT. The coding region from J-TRP (included) to the DONOR-SPLICE of the J-REGION is the FR4-IMGT which corresponds to the G-STRAND of the V-DOMAIN. Thirty-nine labels (27 for “V-GENE” and 32 for “V-D-J-GENE”), of which 20 are shared and twelve relations [51] are necessary and sufficient for a complete description of these prototypes. (With permission from M-P. Lefranc and G. Lefranc, LIGM, Founders and Authors of IMGT^®^, the international ImMunoGeneTics information system^®^, https://www.imgt.org).

Each one of the 21 instances of the ‘Molecule_EntityType’ concept (IDENTIFICATION axiom) is linked to an instance of the ‘Molecule_EntityPrototype’ concept (DESCRIPTION axiom), by the reciprocal relations ‘is_described_by’ and ‘describes’. Each instance of the ‘Molecule_EntityPrototype’ concept is described with its constitutive motifs and IMGT standardized labels [51] (IMGT Scientific chart > IMGT/IMGT standardized labels https://www.imgt.org/ligmdb/label (accessed 14 February 2026) (DESCRIPTION axiom [51]). Prototypes, for example V-J-GENE and V-D-J-GENE (Figure 9) [66], are available on the IMGT^®^ web site (IMGT Scientific chart https://www.imgt.org/IMGTScientificChart/ (accessed 14 February 2026) > 1. Sequence and 3D structure identification and description > IMGT prototypes table).

Prototypes represent the organizational relationship between labels and give information on the order and expected length (in number of nucleotides) of the labels [51]. This provides rules to verify the manual annotation, and to design automatic annotation tools. Annotation of sequences and 3D structures with these labels constitutes the main part of the expertise. IMGT-specific labels defined for sequences are used to describe specific IG and TR gene organization and protein structures. Interestingly, 64 IMGT-specific labels defined for IG and TR nucleotide sequences have been entered in Sequence Ontology (SO) (https://www.imgt.org/IMGTindex/ontology.php) (accessed 14 February 2026) and 41 essays and four chapters on IMGT labels have been published in Encyclopedia of Systems Biology [36,49].

**Figure 10:**
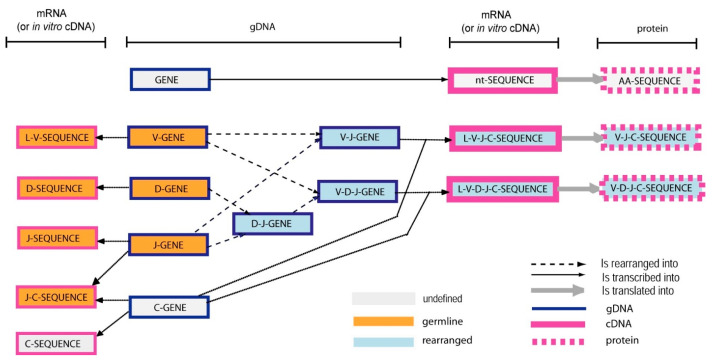
The ‘Molecule_EntityPrototype’ concept of description (DESCRIPTION) [51]. A ‘Molecule_EntityPrototype’ is generated for the description of each Molecule_EntityType. Nineteen entities are defined (labels in capital letters). Three of them, ‘GENE’, ‘nt-SEQUENCE’ and ‘AA-SEQUENCE’, correspond to conventional genes. Sixteen entities allow the description of the IG and TR: six for gDNA, germline (‘V-GENE’, ‘D-GENE’, ‘J-GENE’), undefined (‘C-GENE’), and rearranged (‘V-D-J-GENE’, ‘V-J-GENE’), two for mRNA and for in vitro cDNA (‘L-V-D-J-C-SEQUENCE’, ‘L-V-J-C-SEQUENCE’), two for protein (‘V-D-J-C-SEQUENCE’, ‘V-J-C-SEQUENCE’), one for partial rearrangement (‘D-J-GENE’) and five for sterile germline transcripts, e.g., L-V-SEQUENCE (left column). The ten entities involving IG and TR V, D and J genes show the transition from germline genes to rearranged genes, followed by their transcription into mRNA, and then by the translation into protein sequences. They include V-GENE, D-GENE, J-GENE (orange background), which rearrange into V-J-GENE, D-J-GENE and V-D-J-GENE (blue background) and are transcribed, with the C-GENE (undefined configuration, no background color), into L-V-J-C-SEQUENCE and L-V-D-J-C-SEQUENCE, and translated and processed into V-J-C-SEQUENCE and V-D-J-C-SEQUENCE. (With permission from M-P. Lefranc and G. Lefranc, LIGM, Founders and Authors of IMGT^®^, the international ImMunoGeneTics information system^®^, https://www.imgt.org).

The ‘Molecule_EntityPrototype’ concept is fundamental in IMGT-ONTOLOGY as relations between its instances allow for the representation of the knowledge related to the complex mechanisms of IG and TR gene rearrangements and chain synthesis [1,2,5,6] (Figure 10) [66]. The relation ‘is_rearranged_into’ is specific to the synthesis of the IG and TR. The relations ‘is_transcribed_into’ and ‘is_translated_into’ are general for molecular biology. These three relations allow for the organization of the various instances of the ‘Molecule_EntityPrototype’ concept during the synthesis of the IG and the TR, and in a more general way for the expression of any protein. In addition, by more specific relations, they allow us to take into account the alternative transcripts, the protein isoforms and the post-translational modifications.

More than 500 standardized labels were defined: 221 for the nucleotide sequences (IMGT/LIGM-DB labels https://www.imgt.org/ligmdb/label (accessed on 9 April 2026)) and 285 for the 3D structures (IMGT/3Dstructure-DB labels https://www.imgt.org/IMGTScientificChart/SequenceDescription/IMGT3Dkeywords.html (accessed on 9 April 2026)). A set of 10 relations is necessary and sufficient to compare the localization of the motifs of an instance of the concept ‘Molecule_EntityPrototype’ (Table 3). These relations are part of the concepts of localization (LOCALIZATION axiom) (IMGT Index, https://www.imgt.org/IMGTindex/Localization.php (accessed on 9 April 2026)).

### 3.3. IMGT^®^, the International ImMunoGeneTics Information System^®^: Coherence Between the Components

**Figure 11:**
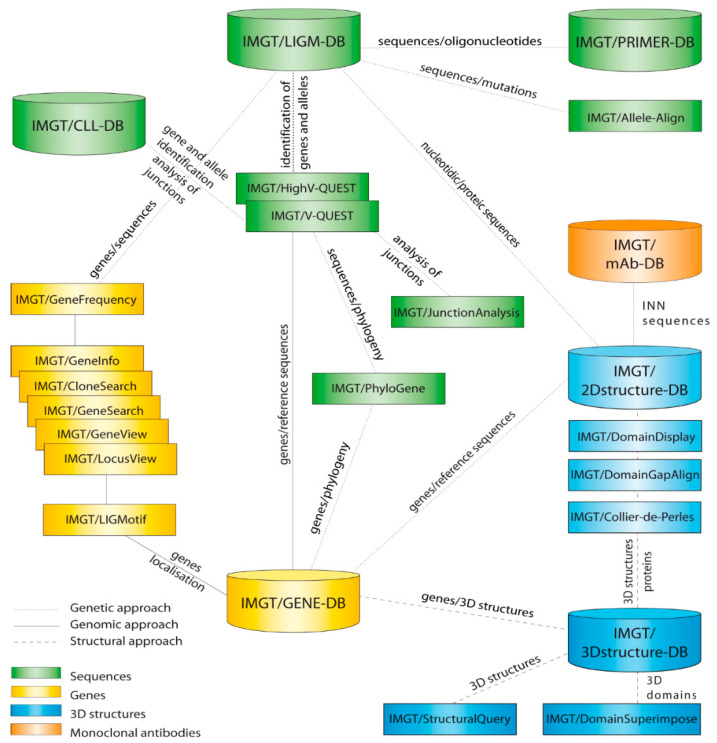
IMGT^®^, the international ImMunoGenetics information system^®^ databases and tools, https://www.imgt.org [1,34,35,36,37,38,39]. IMGT^®^ comprises seven IMGT databases [69,70,71,72,73,74,75,76,77,78] (shown as cylinders), seventeen online IMGT tools [78,79,80,81,82,83,84,85,86,87,88,89,90,91,92,93,94,95,96,97,98,99,100,101,102,103,104,105,106,107] (shown as rectangles) for genes (in yellow), sequences (in green) and structures (in blue), all available from the original IMGT^®^ Home page (Figure 8). IMGT/mAb-DB [77,78] has been online since 4 December 2009. IMGT/HighV-QUEST for next-generation sequencing (NGS) high-throughput sequence analysis, created in October 2010, has been available on the web since 22 November 2010 [78]. (With permission from M-P. Lefranc and G. Lefranc, LIGM, Founders and Authors of IMGT^®^, the international ImMunoGeneTics information system^®^, https://www.imgt.org).

IMGT^®^, the international ImMunoGeneTics information system^®^ [34,35,36,37,38,39], comprises the IMGT databases [69,70,71,72,73,74,75,76,77] and the IMGT tools [78,79,80,81,82,83,84,85,86,87,88,89,90,91,92,93,94,95,96,97,98,99,100,101,102,103,104,105,106,107] represented as cylinders and rectangles, respectively, in Figure 11. Genomic (genes), genetic (sequences) and structural (structures) components are in yellow, green and blue, respectively [34]. Interactions in the genomic, genetic and structural approaches are represented with continuous, dotted and broken lines, respectively [34].

**Figure 12:**
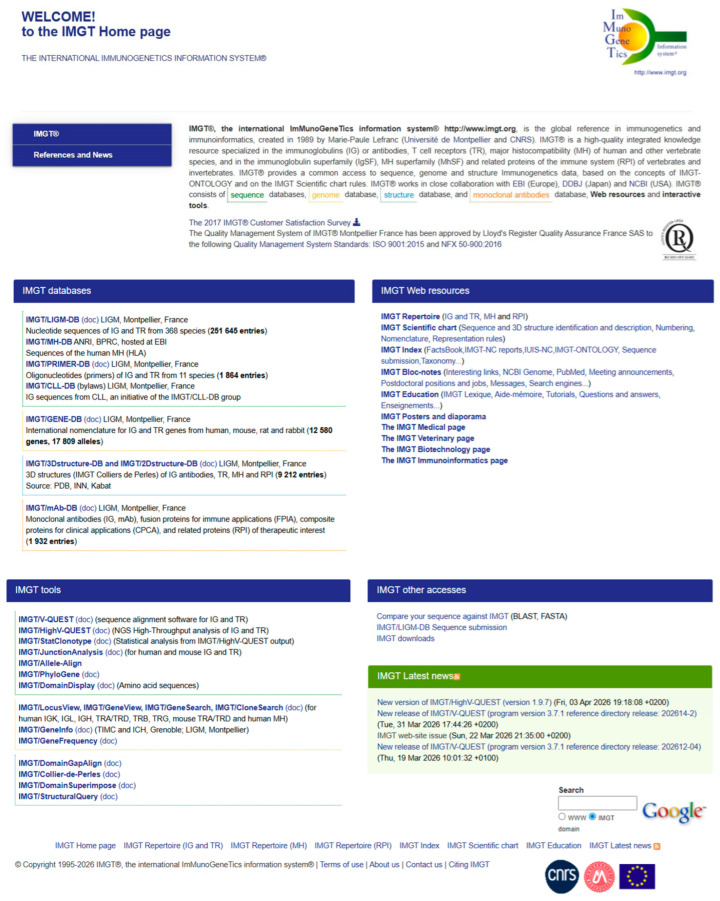
IMGT Home page of the IMGT^®^, the international ImMunoGenetics information system^®^, https://www.imgt.org [1,34,35,36,37,38,39]. The IMGT Home page displays four sections which give direct access to each of the individual seven ‘IMGT databases [69,70,71,72,73,74,75,76,77,78]’, seventeen ‘IMGT tools’ [78,79,80,81,82,83,84,85,86,87,88,89,90,91,92,93,94,95,96,97,98,99,100,101,102,103,104,105,106,107] (with dotted lines in green for sequences, yellow for genes, and blue for structures), ten sections of the ‘IMGT Web resources’ and three ‘IMGT other accesses’, and the ‘IMGT Latest news’.

The original IMGT Home page (Figure 12) displays the four sections ‘IMGT databases’, ‘IMGT tools’, ‘IMGT Web resources’ (more than 25,000 pages, previously the ‘IMGT Marie-Paule page’) and ‘IMGT other accesses’ constitutive of the IMGT^®^, the international information system^®^ (https://www.imgt.org) and gives a direct access to each of the individual seven databases [69,70,71,72,73,74,75,76,77,78], seventeen tools [78,79,80,81,82,83,84,85,86,87,88,89,90,91,92,93,94,95,96,97,98,99,100,101,102,103,104,105,106,107], ten sections of the Web resources [108] or other three accesses. This key feature of the IMGT Home page allows the users, at any time of their queries, to easily return to the IMGT Home page to get direct access to any IMGT database, tool, web resource or other access.

The ‘IMGT databases’ [69,70,71,72,73,74,75,76,77,78] section gives direct access seven databases, three specialized in IG and TR nucleotide sequences (IMGT/LIGM-DB [69], IMGT/PRIMER-DB [70,71], IMGT/CLL-DB [72]), one for IG and TR genes and alleles (IMGT/GENE-DB [73]), and two for amino acid sequence two-dimensional (2D) and three-dimensional (3D) structures (IMGT/2Dstructure-DB and IMGT/3Dstructure-DB [74,75,76]). The IMGT/mAb-DB [77,78] interface allows for the querying of therapeutical monoclonal antibodies (IG, mAb), fusion proteins for immune applications (FPIA), composite proteins for clinical applications (CPCA) and related proteins (RPI) of therapeutic interest (with links to amino acid sequences in IMGT/2Dstructure-DB, and if 3D structures are available, links to IMGT/3Dstructure-DB [74,75,76].

The ‘IMGT^®^ tools’ [78,79,80,81,82,83,84,85,86,87,88,89,90,91,92,93,94,95,96,97,98,99,100,101,102,103,104,105,106,107] include: (1) for nucleotide analysis, IMGT/V-QUEST [79,80,81,82,83,84,98] with the integrated IMGT/JunctionAnalysis [85,86,87,88] and IMGT/Decryption [89] tools and the internal IMGT/Automat [90,91], and for next generation sequencing (NGS), the high-throughput IMGT/HighV-QUEST web portal [78,84,92,93,94,95,98], and the downloadable IMGT/StatClonotype [96,97] package (which allows for statistical pairwise analysis of the diversity and expression of the IMGT clonotypes (AA) and repertoire comparisons in adaptive immune responses); (2) for genome analysis, IMGT/LIGMotif [104] used for the identification and description of new IG and TR genes in large genomic sequences; (3) for amino acid sequence analysis per domain, IMGT/DomainGapAlign [75,78,105,106] and for queries of IMGT/3Dstructure-DB, IMGT/StructuralQuery [74]; and (4) for graphical representation of the domains, IMGT/Collier de Perles [107] (e.g., IMGT Colliers de Perles of the variable (V), constant (C) and groove (G) domains).

The ‘IMGT Web resources’ (previously ‘The Marie-Paule page’) (Figure 12) comprise more than 25,000 pages with direct access to ten sections: IMGT Repertoire (IG and TR, MH, RPI), IMGT Scientific chart (Sequence and 3D structure identification and description, Numbering, Nomenclature, Representation rules), IMGT Index (FactsBook, IMGT-NC reports, IUIS-NC, IMGT-ONTOLOGY, Sequence submission, Taxonomy…), IMGT Bloc-notes (Interesting links, NCBI Genome, PubMed…), IMGT Education (IMGT Lexique, Aide-mémoire (amino acid physicochemical properties [108], splicing sites, Tutorials…), IMGT Posters and diaporama, The IMGT Medical page, The IMGT Veterinary page, The IMGT Biotechnology page, and The IMGT Immunoinformatics page.

The ‘IMGT other accesses’ includes the IMGT/BlastSearch on IMGT sequences databases and the IMGT^®^ downloads (IMGT/LIGM-DB flat files, IMGT/GENE-DB reference sequences in FASTA format, IMGT/3Dstructure-DB flat files, IMGT/V-QUEST reference sequences);

IMGT databases, IMGT tools and examples of items of the IMGT Repertoire (IG and TR) (part of the IMGT Web resources) for the sequences, genes and structures of the IG and TR are listed in Table 4.

The coherence between the components of the system (databases, tools and web resources) is maintained through the use of the standardized IMGT Scientific chart rules (Sequence and 3D structure identification and description, Numbering, Nomenclature, Representation rules) (Figure 12).

## 4. The IMGT Nomenclature (IMGT-NC) of the IG and TR Genes and Alleles

### 4.1. Advent of Immunoinformatics

The V, D, J, and C genes which code the antigen receptors were officially recognized as ‘genes’, as were the conventional genes, at HGM10, in New Haven in 1989, marking the creation of IMGT^®^ and the advent of immunoinformatics [1]. The *Homo sapiens* TRG locus [109] was the first complete antigen receptor locus officially entered in 1989 in the HGM10 database [110,111].

The human (*Homo sapiens*) IG and TR IMGT gene names [2,3,7,8] were approved by the Human Genome Organization (HUGO) Nomenclature Committee (HGNC) in 1999 [112] and entered in the NCBI gene database (first LocusLink, then EntrezGene, and currently Gene, with reciprocal links with IMGT/GENE-DB).

The manual analysis of the germline nucleotide sequences by the LIGM biocuration team has resulted in 25 publications [113,114,115,116,117,118,119,120,121,122,123,124,125,126,127,128,129,130,131,132,133,134,135,136,137], of which 20 were in the section ‘IMGT Locus in Focus’ of Experimental and Clinical Immunogenetics—on human IGL [114], IGK [115], IGH [117,118], TRB [120,123], TRA [121,122], proteins display IG [119], TR [126], mouse IGK [116,134], TRB [129], TRD [130], teleostei IG [124,125], nomenclature human IGH [131], IGK [132], and IGL [133]. Two were in The Immunologist—locus map IG [127] and TR [128]—one was in Developmental and Comparative Immunology—mouse TRA and TRD [136]—and one chapter was in Molecular Biology of B cells on IGL human and mouse [137], with standardization of gene nomenclature, functionality, and allele polymorphism and the setting of the IMGT Scientific chart rules and IMGT unique numbering.

### 4.2. Homo sapiens IG and TR Loci, Genes and Alleles: The Immunoglobulin FactsBook and the T Cell Receptor FactsBook References

IG and TR genes are classified in groups defined by the gene type (V, D, J or C) and by the locus to which they belong. The seven major loci, three for IG (IGH, IGK and IGL) [2,6] and four for TR (TRA, TRB, TRD and TRG) [3], are located on different chromosomes, with the particularity of the TRD locus being nestled inside the TRA locus in higher vertebrates [3]. The princeps publications on the IG and TR loci, genes and alleles of the seven human (*Homo sapiens*) loci are the two FactsBooks published in 2001 [2,3]. The Factsbooks comprise 203 functional and open reading frame (ORF) genes for IG, corresponding to 459 alleles, for a total of 837 sequences [2], and for TR, 168 functional and ORF genes [3]. Entries of the FactsBooks [2,3] provide information on assignment to subgroups and nomenclature, gene definition and functionality, gene location, allelic polymorphism, standardized sequence alignment with protein translation, framework and complementarity determining region (CDR-IMGT) lengths, two-dimensional representations (or Colliers de Perles), IMGT/LIGM-DB and EMBL/GenBank accession numbers, genome database accession numbers (GDB, LocusLink) and key references [2,3]. This information has served as a template for the IMGT Repertoire (IG and TR) https://www.imgt.org/IMGTrepertoire/ (accessed on 9 April 2026)of the IMGT Web resources (the ‘IMGT Marie-Paule page’), which comprises (1) ‘Locus and genes’; (2) ‘Proteins and alleles’; (3) ‘2D and 3D structures’; (4) ‘Probes and RFLP’; (5) ‘Taxonomy’; (6) ‘Gene regulation and expression’; (7) ‘Genes and clinical entities’. Basic IMGT Web resources include ‘Gene tables’, ‘Alignments of alleles’, ‘Protein displays’, ‘Colliers de Perles’, ‘Locus representations’, ‘Potential germline repertoires with CDR-IMGT lengths’, ‘Locus gene order’, copy number variations (CNV) and haplotypes.

This detailed identification, description and classification of the human IG and TR loci, genes and alleles [2,3], using the IMGT Scientific chart rules https://www.imgt.org/IMGTScientificChart/ (accessed on 9 April 2026), is the result of a huge work of annotation and expert analysis, by LIGM, of tens of thousands of nucleotide sequences from phages, cosmids or contigs submitted by the authors to the generalist nucleotide databases (EMBL database, now European Nucleotide Archive (ENA) [138], GenBank [139] and DNA Databank of Japan (DDBJ)) [140]. The annotated sequences were integrated into the then-newly created IMGT/LIGM-DB [14,15], using the EMBL/GenBank/DDBJ accession numbers in order to facilitate interoperability with the generalist nucleotide databases. The ‘Nature’ and ‘Science’ papers on human genome sequencing [141,142], published in 2001, contain limited information on the genes of the antigen receptors of the adaptive immune responses. However a careful analysis of the maps published in these papers allowed us to confirm the chromosomal localizations of the seven main loci—IGH at 14q32.33, IGK at 2p11.2 and IGL at 22q11.2 (for the immunoglobulins), TRA at 14q11.2, TRB at 7q34, TRG at 7p14 and TRD at 14q11.2 (for the T cell receptors)—described in 2001, in the Immunoglobulin FactsBook [2] and in the T cell receptor FactsBook [3], respectively, and determined by an analysis of translocations involving the IG and/or TR loci in leukemia and lymphoma (https://www.imgt.org/IMGTrepertoire/GenesClinical/translocation/human/overview/Hu_overviewpart1.html) (accessed on 14 February 2026).

### 4.3. Extension of the IMGT Nomenclature to Mus musculus and Fish (Chondrichtyes and Teleostei) IG and TR Genes and Alleles

Based on the paradigm of the human loci (IMGT nomenclature, IMGT unique numbering, IMGT standardized keywords and labels), the seven mouse (*Mus musculus*) loci with a total of 625 genes (377 IG and 248 TR) [116,129,130,134,136] were characterized and presented at the 19th International Mouse Genome Conference (IMGC) in 2005 (https://www.imgt.org/IMGTposters/IMGC_IG.html and https://www.imgt.org/IMGTposters/IMGC_TR.html) (accessed on 14 February 2026)), and entered in NCBI Gene, with reciprocal links to IMGT/GENE-DB and in Mouse Genome Informatics (MGI).

The analysis of IG genes in four Chondrichthyes and twenty-two different Teleostei species confirmed that the IG and TR paradigm was applicable for fish; however, most sequences were at that time unmapped and were assigned a provisional nomenclature with the letter S [124,125]. The Chondrichthyes and Teleostei light chain which is neither kappa nor lambda was defined as ‘iota’ encoded by genes of the IG iota (IGI) locus which includes IGIV, IGIJ and IGIC groups (https://www.imgt.org/IMGTrepertoire/LocusGenes/genetable/Teleostei/#IGIV) (accessed on 14 February 2026).

Since 1998, novel genes and alleles of any species have been announced in ‘IMGT^®^ Creations and updates’ after validation by IMGT-NC [67,68].

### 4.4. Management of Genes and Alleles in IMGT/GENE-DB and Corresponding Nucleotide Reference Sequences and Accession Numbers in IMGT/LIGM-DB

Created in 2003, IMGT/GENE-DB [73] provides direct links (access from the Query page) which allow the most frequent requests to be encoded in the form of URL.

A request for a given gene provides (1) the IMGT/GENE-DB entry, (2) the corresponding IMGT/LIGM-DB- nucleotide reference sequence and accession number of each allele of that gene in FASTA format, (3) links to the other IMGT/LIGM-DB sequence(s) with labels in FASTA format, (4) three tables with links to annotated IMGT/LIGM-DB cDNA, to annotated IMGT/LIGM-DB rearranged genomic DNA sequences, and to annotated IMGT/3Dstructure-DB structures and IMGT/2Dstructure amino acid sequences, (5) a table with the gene in genome assemblies with delimitations of the labels of the V-, D-, J- or C-GENE-UNIT.

External links include Nomenclature (HGNC database), Genome databases (NCBI Gene, Ensembl, GeneCards), Protein database (Uniprot), Sequence databases (ENA, DDBJ, GenBank) and for the genome databases, a table in two formats, HTML and CSV format.

On 19 December 2025, IMGT/GENE-DB data include 758 *Homo sapiens* IG and TR genes and 1825 alleles (507 IG genes and 1319 alleles, 251 TR genes and 506 alleles) with links to HGNC, NCBI Gene, Ensembl, GenAtlas, GeneCards and UniProt, and 1228 *Mus musculus* IG and TR genes and 1887 alleles (950 IG genes and 1325 alleles, 278 TR genes and 562 alleles) with links to MGI and NCBI Gene. The information, for each IMGT/GENE-DB entry, include: IMGT gene functionality, IMGT gene definition (for *Homo sapiens* and *Mus musculus* IG and TR), the HGNC gene definition (identical to the IMGT gene definition), number of alleles, chromosomal localization and IMGT/LIGM-DB reference sequence(s) for allele *01. IMGT/GENE-DB is updated weekly, with downloads available in different formats, in the “IMGT downloads” section.

## 5. IMGT-NC Reports for Novel IG and TR Genes and Alleles Names

With the increase in genome sequencing and assembly, the starting point for IG and TR gene identification, description and classification has moved from individual sequences (researchers’ submission to generalist databases) to the IG and TR locus identification in NCBI Whole Genome Assemblies (WGS) (submitted by sequencing groups and analyzed by researchers).

In order to allow researchers to go ahead with expression studies and to publish their data with IMGT gene names even if the loci have not yet been annotated in IMGT^®^ or in other specialist databases, thirty IUIS NOM IMGT-NC Reports have been published from 2017 to 2022 (Supplementary Materials Table S2. List of the IMGT-NC reports 1–30). That initiative has allowed scientists to propose IMGT gene names for new IG and TR variable (V), diversity (D), joining (J) and constant (C) genes and alleles, for a given locus of a given species, based on the IMGT Scientific chart rules and the IMGT-ONTOLOGY concepts of classification (CLASSIFICATION axiom).

**Figure 13:**
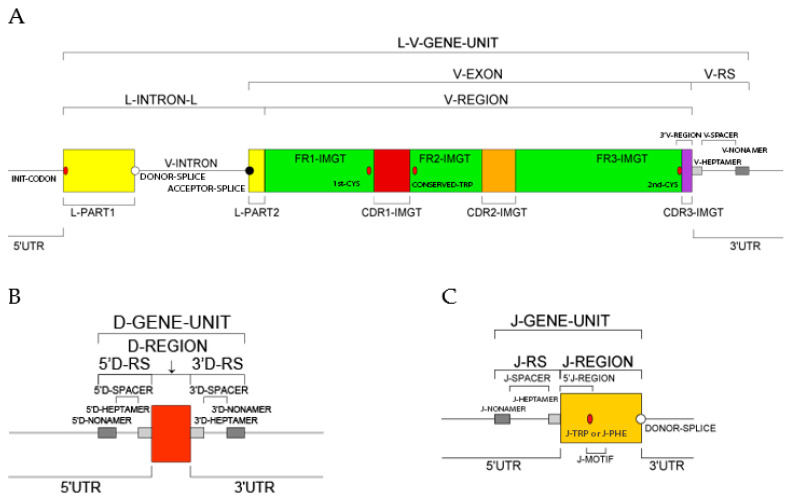
Prototypes with IMGT standardized labels [51]. (**A**) L-V-GENE-UNIT. This label describes gDNA of an IG or TR V-GENE unit, in germline configuration, that comprises L-PART1 (yellow), V-INTRON, V-EXON and V-RS. The V-EXON includes L-PART2 (yellow) and the V-REGION with FR1-IMGT, FR2-IMGT and FR3-IMGT (green), and CDR1-IMGT, CDR2-IMGT and germline CDR3-IMGT (here, red, orange and purple, respectively for an IGH V-GENE https://www.imgt.org/IMGTScientificChart/RepresentationRules/colormenu.php (accessed on 9 April 2026)). (**B**) D-GENE-UNIT. This label describes gDNA of an IG or TR D-GENE unit, in germline configuration, that comprises 5′D-RS, D-REGION (red) and 3′D-RS. (**C**) J-GENE-UNIT. This label describes gDNA of an IG or TR J-GENE unit, in germline configuration, that comprises 5′J-RS and J-REGION (pale orange). Definitions of the IMGT standardized labels are available at https://www.imgt.org/ligmdb/label (accessed on 9 April 2026). Abbreviations: L: leader, RS: recombination signal. (With permission from M-P. Lefranc and G. Lefranc, LIGM, Founders and Authors of IMGT^®^, the international ImMunoGeneTics information system^®^, https://www.imgt.org).

The submission for an IUIS NOM IMGT-NC Report requires that each gene sequence has an accession number in a generalist database (with localization if large original sequence) and that each V, D, J or C gene sequence has been mapped (cloned from bacterial artificial chromosome (BAC), fosmid, cosmid or phage, or extracted from a referenced genome assembly) (Figure 13).

1. For a new V gene and allele, the submitted sequence is that of the L-V-GENE-UNIT: a complete germline genomic sequence (germline gDNA) from the atg (INIT-CODON) of L-PART1 to the V-RS included (https://www.imgt.org/IMGTScientificChart/SequenceDescription/displayimage.php?id=19) (accessed on 14 February 2026) (Figure 13).
2. For a new D gene and allele, the submitted sequence is that of the D-GENE-UNIT: a complete germline genomic sequence (germline gDNA) from the 5′D-RS to the 3′D-RS included (https://www.imgt.org/IMGTScientificChart/SequenceDescription/displayimage.php?id=2) (accessed on 14 February 2026) (Figure 13).
3. For a new J gene and allele, the submitted sequence is that of the J-GENE-UNIT plus DONOR-SPLICE: a complete germline genomic sequence (germline gDNA) from the J-RS to the DONOR-SPLICE included (https://www.imgt.org/IMGTScientificChart/SequenceDescription/displayimage.php?id=9) (accessed on 14 February 2026) (Figure 13).
4. For a new C gene and allele, the submitted sequence is that of the C-GENE-UNIT: a complete genomic sequence (gDNA) from the first codon first exon (EX1) to the STOP-CODON included (this requirement has become effective from 1 January 2018), plus the individual exons, if several (https://www.imgt.org/IMGTScientificChart/SequenceDescription/displayimage.php?id=6) (accessed on 14 February 2026).

The label C-GENE-UNIT describes gDNA of an IG or TR C-GENE unit, in undefined configuration, that comprises exon(s) (EXON), coding the C-REGION, and intron(s) (INTRON) if present, from the first nucleotide of the first exon to the STOP-CODON (included) after the last exon.

Recent examples of veterinary IG and TR loci from genome assemblies, analyzed by scientists using gene and allele names validated by the IUIS NOM IMGT-NC, include: dog (*Canis lupus familiaris*) [143], the first veterinary species with the seven IG and TR loci identified, cat (*Felis catus*) with the four TR loci [144], rabbit (*Oryctolagus cuniculus*) TRA locus [145], dolphin (*Tursiops truncatus*) [146], and salmonid including salmon (*Salmo salar*) and trout (*Oncorhynchus mykiss*) duplicated IGH loci [147,148] and TRA/TRD locus [149]. These examples of different species and loci have been key elements in the setting of the NOM IMGT-NC Reports procedure. They also confirm the necessity for databases using these data (for analysis or biocuration) to cite and link to these original IUIS NOM IMGT-NC reports to guarantee interoperability. For example, it is expected that links to the 30 IUIS NOM IMGT-NC reports are added in IMGT^®^ Creations and updates (https://www.imgt.org/IMGTinformation/creations/ (accessed on 9 April 2026)), on the model of ‘25_IMGT-NC_Report_2021-1-0611_Homsap_IGKV_IGLV’ following data annotation and entry of the new genes and alleles in the IMGT system.

## 6. IMGT-NC New Concepts for Biocuration of IG and TR Loci from Genome Assemblies

### 6.1. Locus in Genome Assembly

**Figure 14:**
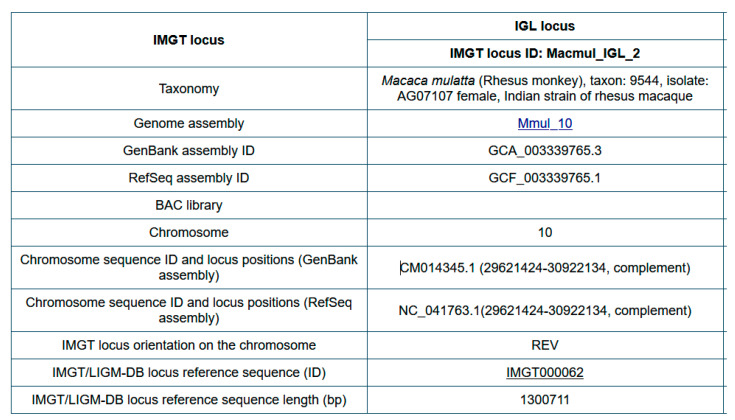
Locus in genome assembly with the example of the IMGT locus ID: Macmul_IGL_2 [150]. Rhesus monkey (*Macaca mulatta*) IGL locus, IMGT Repertoire (IG and TR) 1. Locus and genes. > 3. Locus descriptions > Locus in genome assembly > IGL: Rhesus monkey https://www.imgt.org/IMGTrepertoire/index.php?section=LocusGenes&repertoire=locusAssembly&species=rhesus_monkey&group=IGL (accessed on 14 December 2025). Only the last annotated locus in genome assembly is shown in the figure; annotated loci of previous assemblies validated by IMGT-NC are available online on the right of the displayed locus. (With permission from M-P. Lefranc and G. Lefranc, LIGM, Founders and Authors of IMGT^®^, the international ImMunoGeneTics information system^®^, https://www.imgt.org) (accessed on 14 December 2025).

Before starting IMGT biocuration of a new IG or TR locus of a veterinary species, information is collected in ‘Locus in genome assembly’ (Figure 14).

For an easier comparison between loci of different species, and/or between loci of different genomes assemblies (or of different haplotypes, including CNV), the IDENTIFICATION axiom has been enriched by the implementation of ‘IMGT locus ID’ and ‘IMGT/LIGM-DB locus reference sequence (ID)’ (Figure 14) [150].

### 6.2. IMGT Locus ID and IMGT/LIGM-DB Locus Reference Sequence

An ‘IMGT locus ID’ comprises the 6-letter (or 9-letter) genus and species (or subspecies) Latin names (IMGT taxon abbreviation, e.g., Macmul for *Macaca mulatta*), the locus type (e.g., IGL) and a chronological increasing number, separated by underscores, for example, Macmul_IGL_2 (Figure 14) [150]. Hyphens instead of underscores may be used in text, e.g., Macmul-IGL-2.

An ‘IMGT/LIGM-DB locus reference sequence’ is an IMGT accession number (‘IMGT’ followed by six digits) which identifies the IMGT/LIGM-DB flat files containing an IG or TR locus (or part of it) extracted from an NCBI genome assembly and presented in its own 5′ to 3′ locus orientation [150]. As a locus may have, on the chromosome, a forward (or ‘Watson’) (FWD) or a reverse (REV) orientation (IMGT Index > Genomic orientation), the sequence orientation in the IMGT accession number flat file is either unchanged (direct) relative to the sequence on the chromosome for an FWD locus, or reverse complemented for a REV locus. For example, the rhesus macaque (*Macaca mulatta*) IGL locus orientation on chromosome 10 is reverse (REV) and the IMGT/LIGM-DB locus reference sequence in IMGT000062 is reverse-complemented relative to the sequence on chromosome 10.

The information from ‘Locus in genome assembly’ (Figure 14) is reported in the definition lines (DE) of the IMGT/LIGM-DB locus reference accession number. For IMGT000062 it includes: Macaca mulatta (Rhesus monkey), taxon:9544, isolate: AG07107 single Indian origin rhesus female, assembly Mmul_10, 2345051 [UID], GenBank assembly ID: GCA_003339765.3, Refseq assembly ID: GCF_003339765.1, chromosome 10: CM014345.1 (29621424-30922134, complement), IMGT locus ID: Macmul_IGL_2.

### 6.3. IMGT-LOCUS-UNIT Label and Qualifiers

The label IMGT-LOCUS-UNIT (DESCRIPTION axiom) was created to describe a locus, isolated from a genome assembly, in an IMGT accession number flat file. The definition of the IMGT-LOCUS-UNIT and its qualifiers are given in Table 5.

Three IMGT/LIGM-DB locus reference sequences for the *Macaca mulatta* (rhesus monkey) IGH (IMGT000064, TPA: BK063715), IGL (IMGT000062, TPA: BK063717,) and IGK (IMGT000063, TPA: BK063716) were created and annotated for the Mmul_10 assembly (GCF_003339765.1).

As an example, the IMGT000062 qualifiers for the IMGT-LOCUS-UNIT (1..1300711) of the Macmul_IGL_2 (IMGT_locus_ID) [150] are the following:

FT/IMGT-LOCUS-UNIT 1..1300711

FT/IMGT_locus_3prime_borne = “RSPH14”

FT/IMGT_locus_3prime_gene = “IGLC7”

FT/IMGT_locus_5prime_borne = “TOP3B”

FT/IMGT_locus_5prime_gene = “IGLV(IV)-127”

FT/IMGT_locus_ID = “Macmul_IGL_2”

FT/IMGT_locus_chromosome = “10”

FT/IMGT_locus_length = “1300711 bp”

FT/IMGT_locus_name = “Macaca mulatta IGL”

FT/IMGT_locus_orientation = “reverse (REV)”

FT/IMGT_locus_positions = “CM014345.1

FT (29621424-30922134 complement)”.

### 6.4. IMGT Locus 5′ and 3′ Bornes

The IMGT Locus 5′ borne (IMGT_locus_5prime_borne) and the IMGT Locus 3′ borne (IMGT_locus_3prime_borne) (Table 5) are defined for a standardized comparison of the IG and TR locus delimitation across species [150]. The IMGT bornes are genes coding for a protein (other than IG or TR), conserved between species, located upstream of the first gene (for the IMGT 5′ borne) or downstream of the last gene (for the IMGT 3′ borne) of an IG or TR locus (https://www.imgt.org/IMGTrepertoire/LocusGenes/bornes/bornesIGH.html (accessed on 9 April 2026), IMGT Repertoire (IG and TR) > 1. Locus and genes > 3. Locus descriptions > Locus bornes: IGH, IGK, IGL, TRA, TRB, TRG, TRD). If IMGT bornes are not yet identified or are too distant to be included in the locus sequence, a minimal 10 kb sequence is added upstream of the first IG or TR gene in 5′ and/or downstream from the last IG or TR gene in 3′. An overview of the locus IG and TR 5′ and 3′ bornes is shown in Table 6.

### 6.5. IMGT/GENE-DB Localization in Genome Assemblies

**Figure 15:**
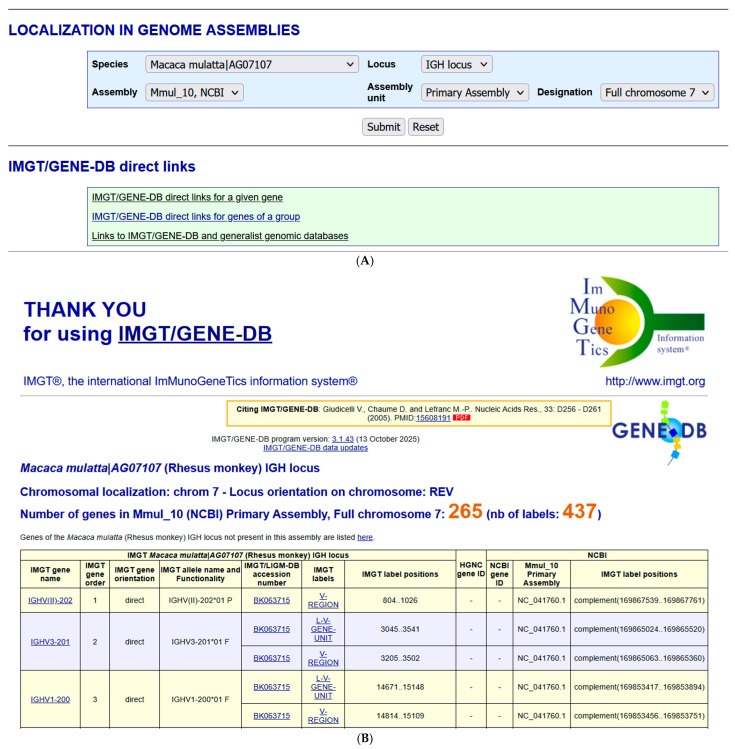
IMGT/GENE-DB Localization in genome assemblies [150]. (**A**) Query for Macaca mulatta|AG07107 (Species, AG07107 isolate) and IGH (Locus) showing the availability of IMGT/GENE-DB biocurated genes for the assembly ‘Mmul_10, NCBI’. (**B**) Top of the results page for the query. IMGT alleles of a given gene are defined by the number which follows the asterisk (i.e., *01). The alternating colors are for better readability. (With permission from M–P. Lefranc and G. Lefranc, LIGM, Founders and Authors of IMGT^®^, the international ImMunoGeneTics information system^®^, https://www.imgt.org) (accessed on 14 December 2025).

The section “LOCALIZATION IN GENOME ASSEMBLIES” (Figure 11) integrated in 2015 in IMGT/GENE-DB allows, for a given species and a given locus, to query the IMGT IG or TR genes of a given genome assembly. The query Species: Macaca mulatta|AG07107 (AG07107 is the isolate) and Locus: IGH locus shows the availability of IMGT/GENE-DB biocurated genes for the assembly ‘Mmul_10, NCBI’, ‘Primary Assembly’ ‘Full chromosome 70 [150] (Figure 15A).

The results page for the query Species: Macaca mulatta|AG07107 (Rhesus monkey) Locus: IGH (Figure 15B) provides at the top the chromosome localization: ‘chrom7’, the locus orientation on chromosome ‘REV’, the number of genes in Mmul_10 (NCBI) (IMGT/GENE-DB annotated genes) ‘265’ and the number of labels ‘437’. For each gene, the information comprises IMGT gene name, IMGT gene order, IMGT gene orientation (direct (5′ > 3′) or opposite (3′ > 5′) in the locus), IMGT allele name and Functionality (F, ORF or P), IMGT/LIGM-DB accession number, IMGT labels (L-V-GENE-UNIT and V-REGION for a V gene, D-GENE-UNIT and D-REGION for a D gene, J-GENE-UNIT and J-REGION for a J gene, C-GENE-UNIT and C-REGION or the different individual exons for a C gene), IMGT label positions in the IMGT/LIGM-DB accession number, HGNC gene ID (for *Homo sapiens*), NCBI gene ID (if available), NCBI Mmul_10 Primary Assembly Chromosome (NC) accession number and IMGT label positions in the NC sequence.

The list of genes known to belong to the locus but not localized (NL) in the assembly is also provided in this section as this may correspond to polymorphism by copy number variation, insertion/deletion, or gaps in the assembly.

## 7. IMGT-NC New Concepts for Gene Copy Number Variations (CNV): ‘CNV Haplotypes’ and ‘CNV-Locus-Haplotype’

### 7.1. IMGT-NC Gene Copy Number Variation (CNV) Nomenclature and Definition

IMGT-NC gene copy number variation (CNV) nomenclature and definition are illustrated with the *Homo sapiens* IGH locus [2,5,150,151]. Gene copy number variations (CNVs) [150] are numbered from 5′ to 3′ in the locus (IMGT Repertoire (IG and TR) > Locus gene order > Human (*Homo sapiens*) IGH). Seven CNV are displayed in Table 7, six for the IGHV genes (CNV1 to CNV6) and one (CNV7) for the IGHC genes [150].

The IMGT CNV haplotype nomenclature comprises the genus and species (Latin names in italics) (e.g., *Homo sapiens*), the locus (e.g., IGH) and the CNV number (e.g., CNV1). A CNV is delimited by a 5prime gene and a 3prime gene; for example, for the *Homo sapiens* IGH CNV1 haplotype, the CNV1-5prime gene is IGHV2-70 (F, ORF, 16 being the gene order) and the CNV1-3prime gene is IGHV1-69 (F,21) (Table 7) [150].

The IMGT standardized definition of a CNV comprises the group, then between parentheses the order of the start gene (the gene which follows the CNV-5prime), a dash and the order of the end gene (the gene which precedes the CNV-3prime), then the total number of genes involved in the CNV (between the 5prime and 3prime, including RPI gene(s) if present) followed, between parentheses, by the number of IG or TR per functionality (and the number of RPI if present). For instance, the definition of ‘*Homo sapiens* IGH CNV1’ is ‘IGHV(17–20)7(3F,4P)’ (Table 7) [150]. The letters ‘i’ for insertion, ‘d’ for deletion, and ‘e’ for exchange may be added if needed to indicate the status of a given CNV or that of given gene(s) or groups of genes in a given haplotype by comparison to the haplotype A (e.g., ‘CNV5’ split in ‘IGHV-e1(145–147)’ and ‘IGHV-e2 (148–149.2)’ [150] (Table 7)). The information on gene presence or absence, or insertion/deletion, is provided with colors (orange: genes present in haplotype A (including CNV-5prime and CNV-3prime) and in other haplotypes of a given CNV; pale green: genes absent in haplotype A and other haplotypes, but present by insertion in other haplotype(s) of a given CNV; red: genes absent, by comparison to haplotype A of a given CNV; green: genes present, as insertion by comparison to haplotype A of a given CNV [150] (Table 7)).

### 7.2. IMGT CNV Haplotypes Illustrated with the Homo sapiens IGH Locus

The description of each CNV haplotype is achieved by comparison with the IMGT Locus representation of the FacstBooks published in 2001 [2,3] (IMGT Repertoire (IG and TR) > 1. Locus and genes > 2. Locus representation > IGH: Human). The genes, localized on the horizontal main line which represents the locus from 5′ to 3′, conventionally, define the ‘haplotype A’ of each individual CNV. A well-characterized CNV example is the *Homo sapiens* IGH CNV3 IGHV(87–112)26(8F,16P,2RPI), for which six haplotypes A to F [3,150] correspond to polymorphic amplifications of genes, found in individuals of different populations. These polymorphisms are described as a multiple of insertion/deletion between IGHV4-34 (86, CNV3-5prime) and IGHV4-28 (113, CNV3-3prime) (Table 7). The longest haplotype (CNV3 C, 26 genes, 98 kb) corresponds to eight motifs (‘4 pairs of motifs’ or ‘quadruplication’) which each contains three IGHV genes: the first gene of each motif is functional (IGHV4 subgroup gene (green in the right column), starting from 5′prime IGHV4-34, in alternance with IGHV3 subgroup gene (blue in the right column) for the second motif of the pair), followed by a pseudogene of the IGHV3 subgroup and a pseudogene of the IGHV(II) clan. The motifs 2 (IGHV3-33) and 8 (IGHV3-30) are characterized by the presence of GOLGA4P1 and GOLGA4P2 (yellow in the right column), respectively, with GOLGA4P1 containing an Alu sequence amplified in the IGH locus (M.-P. personal communication). The haplotype CNV3 C was built from Han Chinese KC162924, KC162925, AC244456, AC231260 contigs with a detailed IMGT nomenclature and biocuration of the pseudogenes which highlighted the eight paired motifs (or ‘quadruplication’). The graphical representation of the CNV3 haplotypes is available at ‘Human (*Homo sapiens*) Polymorphism by insertion/deletion between IGHV4-34 and IGHV4-28 (haplotypes A to F) on chromosome 14 (14q32.33)’ (https://www.imgt.org/IMGTrepertoire/index.php?section=LocusGenes&repertoire=locus&species=human&group=IGH/haplotypes#locus) (accessed on 15 December 2025). The haplotype CNV3 C has been observed in T2T-CHM13v2.0 assembly (24 janvier 2022, GCA_009914755.4, hg38). The haplotypes CNV3 A (AB019439, GRCh37) and CNV3 B (AC245166), with 14 genes (50–55 kb), correspond to four motifs (‘2 pairs of motifs’ or ‘duplication’). The haplotype CNV3 D (AC244464, Han Chinese) with seven genes (25 kb) is the smallest haplotype with two motifs (‘1 pair of motifs’). The CNV3 E (20 genes) and CNV3 F (Yoruba, 19 genes) (70–75 kb) correspond to six motifs (‘3 pairs of motifs’ or ‘triplication’).

The *Homo sapiens* IGH locus on chromosome 14 (14q32.33) is characterized by a remarkable IGH CNV, the CNV7 IGHC(203–211)9(7F,1OP,1P) with seven haplotypes A to G (Table 7), with six of them (haplotypes B to G) corresponding to multigene deletions I to VI, identified on both chromosomes 14 in healthy individuals lacking several subclasses [2,5,150]. Multigene deletions of haplotypes B to G (either identical or different, on both chromosomes in a given individual) are designated I to VI according to the chronological order in which they were found (reviewed in [5]). Deletion I, first identified by the absence of the Gm1 allotypes in a 70-year-old healthy Tunisian woman (TAK3), homozygous for that deletion [152,153], allowed for the ordering of the *Homo sapiens* IGHC genes in the IGH locus [154,155]. Deletions I and II [152,153,156] (haplotypes IGH CNV7 B and C), found in healthy individuals from consanguineous families, involve highly homologous spots of recombination [157], as also described in a healthy individual (T17) homozygous for deletion III (haplotype IGH CNV7 D) and lacking IgA1, IgG2, IgG4 and IgE [158].

The IGHC duplications comprising the duplication of the IGHG4A in 5′ of IGHG4 (gene order 208.1) (CNV7 H) and the multigene duplications including IGHGP (206.1), IGHG2 (206.2), IGHG4 (206.3), IGHE (206.4), and one IGHA (206.5) are still in progress and not detailed here.

### 7.3. IMGT Locus CNV-Haplotype: The Example of the IMGT IGH Locus CNV1-7-Haplotype

With its seven CNV haplotypes, the *Homo sapiens* reference IGH Locus [2] has, by convention, an IMGT Homsap IGH Locus CNV1-7-Haplotype ‘A.A.A.A.A.A.A’, characterized by the haplotype A of the seven CNV involved in the analysis. It is the one obtained from the IGH locus and genes from contig sequences available in the generalist databases and annotated by LIGM in IMGT/LIGM-DB with the Locus representation published in 2001 [2] (Table 8A). The availability of genome assemblies has made it possible to characterize the expected differences in terms of CNV (Table 8B). The ‘IMGT IGH Locus CNV1-7-Haplotype’ from GRCh38.p12 is ‘B.A.B.A.B.B.A’ where the CNV5 B haplotype corresponds to BAC clone sequences [151] from the CHORI-17 BAC library. The ‘IMGT Homsap IGH Locus CNV1-7-Haplotype’ from T2T-CHM13v2.0 is ‘A.A.C.A.A.B.H’ with the duplicated IGHG4A gene, whereas the ‘IMGT Homsap IGH Locus CNV1-7-Haplotype’ from GRCh38.p14 is ‘A.A.A.A.A.A.A’. This has set up the grounds for standardized analysis and comparison of haploid, maternal or paternal chromosomal assemblies.

The CNV representations and the CNV characterization of the IGH, IGK and IGL loci as well as those of the TRA/TRD, TRB and TRG [150] are useful to compare the diversity and polymorphism of the IG and TR loci between individuals of the same species, here *Homo sapiens* [150], but also to study the evolution of the loci of closely related species, such as *Gorilla gorilla gorilla* where the same gene order as *Homo sapiens* can be used. The analysis of IMGT locus CNV-haplotypes which are partial owing to a V-(D)-J rearrangement may provide, for information, the genes and alleles names of the rearranged V, (D) and J genes with their respective gene order, in place of the first missing CNV haplotype. 

## 8. IMGT-NC New Concepts of Engineered IGHG Variants for Effector Properties, Half-Life and Structures of Therapeutic Antibodies

The constant region of the immunoglobulin (IG) or antibody heavy gamma chain is frequently engineered to modify the effector properties of the therapeutic monoclonal antibodies, their half-life and/or their format and structure [159]. The standardized IMGT engineered variant nomenclature [159] has been set up for an easier comparison between engineered antibody variants involved in effector properties (ADCC, ADCP and CDC), half-life and structure of therapeutical monoclonal antibodies [159,160]. The IMGT nomenclature of the IGHG variants is based on a classification in four categories of ‘effector’, ‘half-life’, ‘physicochemical’ and ‘structure’, and in 18 types, assigned to one of the four categories, depending on their property and function types [159]. The ‘effector’ category comprises eight types (1–8): 1. ADCC reduction, 2. ADCC enhancement, 3. ADCC and ADCP enhancement, 4. CDC enhancement, 5. CDC reduction, 6. ADCC and CDC reduction, 7. FcγRIIB binding increase and B cell inhibition (coengagement of antigen and FcγR on the same cell), 8. knock out CH2 84.4 glycosylation (ADCC reduction). Two categories comprise a single type: the ‘half-life’ category includes the type 9 (half-life increase or decrease), whereas the ‘physicochemical’ ‘category’ includes the type 10 (abrogation of binding to Protein A, thermal stability, pI, reduced acid-induced aggregation). The ‘structure’ category comprises eight types (11–18): 11. formation of additional bridge for domain stabilization including scFv, 12. prevention of IgG4 half-IG exchange, 13. hexamerization, 14. knobs-into-holes and enhancement of heteropairing H-H of bispecific antibodies, 15. suppression of inter H-L and/or inter H-H disulfide bridges, 16. site-specific drug attachment, e.g., additional cysteine, 17. enhancement of heteropairing H-L of bispecific antibodies, 18. control of half-IG exchange of bispecific IgG [159,160] (Table 9).

The IMGT engineered variant characterization (Table 10) comprises:

(1) The IMGT engineered variant name which comprises the species (e.g., Homsap for *Homo sapiens*), the variant type(s) (number(s) from 1 to 18), the gene name abbreviation (e.g., G1 for IGHG1), the letter ‘v’ with a number (e.g., Homsap 1-G1v1).
(2) The IMGT engineered variant definition which comprises, for each engineered AA change, the domain (e.g., CH1, CH2 or CH3) or the hinge, the AA in the one-letter abbreviation [108] with its position according to the IMGT unique numbering for C domain [56], followed by the Eu-IMGT position between parentheses, e.g., Homsap 1-G1v1, CH2 P1.4 (233) [159,160]. In the World Health Organisation International Nonproprietary Name (WHO INN) description of therapeutic antibodies [161,162], the Eu-IMGT position is replaced by the position of the AA change in the antibody chain sequence.
(3) The IMGT AA change(s) with the Eu-IMGT position(s) between parentheses (e.g., CH2 **P**114 > **A** (329)).
(4) The AA change(s) at the Eu-IMGT position(s) (e.g., P329A).
(5) The IMGT topological motif sequence identifiable in gene and domain with positions according to the IMGT unique numbering [56], followed, between parentheses, by the Eu-IMGT positions; the display of the motif shows the AA involved in the change highlighted in bold, with red before the change and green after the change, respectively (e.g., IGHG1 CH2 1.6–3 AP**E**LLGGPS > AP**P**LLGGPS; underlined amino acids in the motif correspond to additional positions in the IMGT unique numbering for the C-domain [56], e.g., APELLG and APPLLG which correspond to 1.6, 1.5, 1.4, 1.3, 1.2 and 1.1).
(6) ‘Property and function’ (at least one and up to three per variant) and 3D structure if available [159,160].

## 9. IMGT-NC for Common Scientific Research Endeavor

### 9.1. IMGT-NC a Foundational and Unifying Infrastructure for Immunogenetics and Immunoinformatics

The IMGT nomenclature of the IG and TR gene and allele names, together with the IMGT nomenclature of CNV and haplotypes [150,151,152,153,154,155,156,157,158], the IMGT nomenclature of engineered IGHG variants involved in antibody effector properties and formats [159,160], used in the World Health Organisation International Nonproprietary Name (WHO INN) description of therapeutic antibodies [161,162], and the WHO/IMGT nomenclature of the allotypes [163,164], which bridges serology and proteomics [165,166,167,168,169,170,171,172,173], contribute with the IMGT unique numbering [66] (for V domain [55], C domain [56]) to the common language for IG and TR genes, sequences and structures, as exemplified in antibody engineering and humanization, structural immunology, and therapeutic antibody development [35,36,38,174,175,176,177,178,179,180,181,182,183]. The IMGT numbering has been extended to the groove (G) domain [57] of the major histocompatibility (MH) proteins and MH superfamily (MhSF). They allow for a standardized comparison of IG/Ag complexes, TR/pMH1,/pMH2 and/pMH1-like complexes [66,183,184], including TR-mimic Fab/pMH1 complexes [66,183].

### 9.2. IMGT Nomenclature for Salmonid IG and TR (Pierre Boudinot, Susana Magadán)

The emergence of high-throughput sequencing in the mid-2000s created new opportunities to investigate IG/TR loci evolution in under-investigated species, such as teleost and reptilian. In early studies aiming to identify immunoglobulin isotypes rather than to produce comprehensive germline reference sets, the IMGT system was instrumental for the resolution of IGHC gene content, exon structure, and isotype diversification in newly sequenced genomes. This was especially important to determine orthology relationships between divergent teleost or reptilian sequences and mammalian isotypes [185,186,187,188,189,190,191]. IMGT-ONTOLOGY and standardized feature annotation facilitated accurate exon definition, identification of conserved Cys/Trp motifs, and characterization of structural features such as hinge regions.

A comprehensive description of loci later became necessary to analyze expressed repertoires in species like rainbow trout and Atlantic salmon. A standardized IG/TR gene nomenclature was then generated by the groups of Pierre Boudinot at the University Paris-Saclay, INRAE (France) and of Susana Magadán at the University de Vigo (Spain) that could be updated and reused by the scientific community [147,148,149,192,193]. IMGT provided a standardized, ontology-driven framework that allowed accurate annotation from reference genome sequences of Salmonids, and efficient definition of VDJ gene nomenclature. Using a consistent set of gene definitions (e.g., what constitutes V subgroups, as well as VDJC genes/alleles) and feature boundaries (leader, FR/CDR, recombination signals (RS), splice sites), a consistent description of the architecture of *Oncorhynchus* rainbow trout and Atlantic salmon IGH/TR loci was established and published [147,148,149,193]. Particularities of salmonid IGH/TR loci, such as multiple loci in each haplotype and a large number of genes and pseudogenes, were taken into account to create a nomenclature consistent with the complexity of germline repertoires of these species. The IMGT/HighV-QUEST [92,93,94,95,96] was instrumental in the analyses of expressed repertoires in these species [194,195,196,197,198,199,200,201,202,203,204]. The setting of the IMGT nomenclature for Salmonid required (i) knowledge of the bases for analysis and annotation of VDJ genes, early definition of subgroups and definition of rules for nomenclature (germline/potential repertoires) [147,148,149,185,186,187,188,189,190,191,192,193], and has allowed (ii) constitution of the germline reference sets used as a resource for annotation of expressed repertoires [194,195,196,197,198,199,200,201,202,203,204]. This double goal serves as a model for any non-canonical species and opens novel perspectives on repertoire analysis [205].

### 9.3. IMGT Nomenclature for Less Canonical Antigen Receptors (Michael Criscitiello)

The standardized lingua franca provided by the IMGT is indispensable for work in traditional model species. For the group of Michael Criscitiello at the Texas A&M University (USA) [192,206,207,208,209,210,211,212,213,214,215,216,217,218,219], the curation has been just as necessary when trying to describe less canonical antigen receptors such as cattle immunoglobulin ultralong CDR3H V(D)J junctions [206] or shark loci combining both B and T cell receptor components [213]. The established ground maintained by the IMGT nomenclature gives a firm launch site into discussion of the more divergent and debatable loci that vertebrates have evolved, and we can exploit in antibody and immunotherapeutic engineering. ‘Nomenclature is sometimes viewed as an encumbrance, an onerous chore to move away from the parlance you were taught and adopt someone else’s. I appreciate that others helped me appreciate the need for standard language in communicating complex topics such as antigen receptor loci immunogenetics’. Michael Criscitiello soon became an advocate for and a member of the IUIS-NOM IMGT IG, TR and MH nomenclature committee (IMGT-NC) and worked with the group in efforts to clarify model species loci from zebrafish (*Danio rerio*) TR [208], to Florida manatee (*Trichechus manatus latirostris*) IG [209], to bovid ultralong CDR3 components [217], to pinniped California sea lion (*Zalophus californianus*) MH [219], to teleost IG isotypes [192].

### 9.4. IMGT Nomenclature for Mammalian TR (Salvatrice Ciccarese)

**Figure 16:**
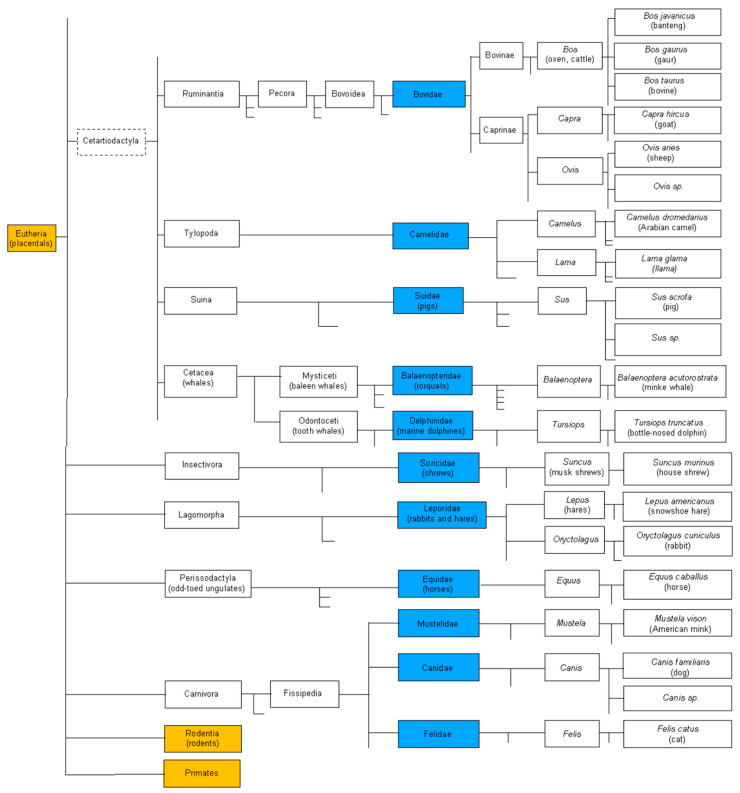
IMGT taxonomy tree: Eutheria (placentals). The “Cetartiodactyla” NCBI taxonomy level of classification groups together the previously designated “Artiodactyla” and the “Cetacea”. A dotted rectangle indicates that the corresponding NCBI taxonomy level of classification is not mentioned in IMGT flat files (and therefore cannot be queried in IMGT/LIGM-DB). A non-limited vertical line indicates that there are more than 5 subdivisions. Available IMGT taxonomy detailed trees are in orange. Families are in blue. https://www.imgt.org/IMGTrepertoire/Taxonomy/vertebrates/eutheria.html (accessed on 9 April 2026). (With permission from M-P. Lefranc and G. Lefranc, LIGM, Founders and Authors of IMGT^®^, the international ImMunoGeneTics information system^®^, https://www.imgt.org).

Investigations on mammalian T cell receptors αβ and γδ, conducted in the group of Salvatrice Ciccarese of the University of Bari Aldo Moro (Italy) using the IMGT nomenclature, have progressed through several key stages [146,220,221,222,223,224,225,226,227,228,229,230,231,232,233,234,235,236,237,238,239,240,241,242,243,244,245,246,247,248,249,250,251,252,253]. An initial step was the genomic mapping of the TRGV–TRGJ–TRGC and TRDV–TRDJ–TRDC genes in Bovidae (Figure 16). This analysis revealed the splitting of the TRG locus into two distinct loci, a structural peculiarity of this taxonomic group.

The duplication was clearly demonstrated in *Ovis aries* (sheep) through BAC clone analysis [220,221,222,223,224,225,226,227,228,229]. Subsequent comparison of the TRG locus organization in Bovidae with the corresponding deduced structure in *Canis lupus familiaris* (dog) [230], *Sus scrofa* (pig) [249], *Equus caballus* (horse) and *Equus asinus* (donkey) species [251] provided important insights into the evolutionary dynamics of the gamma locus in mammals [245]. A further step involved the analysis of spleen cDNA minilibraries from *Camelus dromedarius* (dromedary). This study highlighted the occurrence of somatic hypermutation in rearranged TRGV and TRDV genes [232,233,237]. The use of IMGT tools for the analysis of amino acid sequences deduced from spleen transcripts was instrumental in defining both the timing and the modalities of somatic mutation onset. Attention then shifted to the genomic organization and evolutionary analysis of the beta locus (TRB) [234,236], which revealed shared evolutionary patterns among Tylopoda, Ruminantia and Suina. Within the Cetartiodactyla superorder, the characterization of the TRB locus [146], together with the organization of the TRA/TRD and TRG loci in the marine mammal *Tursiops truncatus* (common bottlenose dolphin) [239], further refined our understanding of the evolutionary landscape of T cell receptor loci. Finally, CD1D-restricted γδ T cell receptors were identified through three-dimensional structural modeling based on *C. dromedarius* cDNA clones [250]. Protein–protein interactions were analyzed within a TRG–TRD/CD1D complex selected for camel-specific features of antigen receptors, including long CDR3-IMGT regions and the presence of somatic mutations [253]. The IMGT numbering of V [55], C [56] and G [57] domains provided a unifying framework to bridge amino acid sequences, 3D models, structures and functions across species [254,255,256,257].

### 9.5. IMGT Nomenclature for IG and TR Gene Repertoires in Lymphoproliferative Disorders (Kostas Stamatopoulos)

Investigations of IG and TR gene repertoires in lymphoproliferative disorders using IMGT nomenclature started as early as 2002 under Kostas Stamatopoulos’s initiative at the Centre for Research and Technology Hellas (Greece) and Karolinska Institutet (Sweden) and have continued uninterruptedly ever since [258,259,260,261,262,263,264,265,266,267,268,269,270,271,272,273,274,275,276,277,278,279,280,281,282,283,284,285,286,287,288,289,290,291,292,293,294,295,296,297,298,299]. The first critical step concerned the collaboration between IMGT and ERIC, the European Research Initiative on CLL, with the main aim of developing and maintaining a database with immunogenetic data from patients with Chronic Lymphocytic Leukemia (CLL) attended at academic institutions, to foster high-quality collaborative research on this disease and related disorders. This initiative led to the establishment of IMGT/CLL-DB (https://www.imgt.org/CLLDBInterface/query (accessed on 9 April 2026)), the largest IG gene sequence database globally, currently holding data from more than 80,000 patients with CLL from 54 institutions from all over the world. IMGT/CLL-DB has facilitated several large-scale immunogenetic research projects focused on unraveling the IG gene repertoire in CLL with important biological and clinical implications, leading to several high-impact and highly cited publications [261,264,265,266,267,277,278,288].

The use of the IMGT nomenclature in the collaboration between ERIC and IMGT has been instrumental in advancing the standards of routine diagnostics in CLL. This has been achieved through the continuous refinement of software (indicatively, allowing for accurate annotation of insertions and deletions in rearranged IG genes); prime educational events (hands-on international workshops organized biannually); and recommendations about the analysis of IG gene rearrangements in both a research and a diagnostic setting [275,276,300]. Importantly, these standards are widely used by the global community of scientists and healthcare professionals working on CLL.

Studies of lymphoproliferative disorders enabled by/relying on IMGT nomenclature in software and tools extend beyond CLL to other entities. Indicative examples are offered by large-scale multi-institutional, multi-national projects on the IG gene repertoires of, e.g., mantle-cell lymphoma [258,260], splenic marginal-zone lymphoma [262,263,269,298] and other marginal-zone lymphoproliferations [259,281], amongst others.

Another major area of interest in lymphomas concerns the analysis of bystander T cells in the tumor microenvironment. Also in this context, IMGT nomenclature used in the processing of big datasets originating from high-throughput, next generation sequencing projects have been fundamental to improved molecular characterization of the TR gene repertoire, laying the ground for novel immmunotherapeutic strategies [268,273,285,291].

## 10. Concluding Remarks

The IMGT nomenclature supports primary immunodeficiency diagnosis, minimal residual disease tracking, vaccine response studies, cancer immunogenetics, and therapeutic antibody design. The major challenge of immunogenetics is how to meet the highest standards and ensure consistency that translates into obvious benefits for both advancing research and providing accurate diagnostic information and helping to find the best and most appropriate therapeutics [300]. The IMGT nomenclature brings a foundational, unifying infrastructure to immunogenetics and immunoinformatics [1] and has made the huge genetic diversity of the IG and TR antigen receptors standardized, computable, and comparable worldwide. It has become the global reference for the IG and TR because it provides a rigorous, universal, and biologically meaningful framework to identify, describe and classify the IG and TR genes, sequences and structures. The standardized IMGT unique numbering for structures is used for AI search [301,302,303,304,305,306,307,308,309,310]. The IMGT nomenclature is used for any IG and TR genes from *Homo sapiens* [296,311,312] and from any other jawed vertebrate species, enabling comparative immunology, evolutionary studies of immune systems, clinical, veterinary and zoonotic research, and evolutionary immunogenetics. By disseminating the standardized IMGT Scientific chart rules used worldwide to name genes, researchers participate in a global scientific research endeavor for the development of immunogenetics and immunoinformatics with the use of AI. In this context, speaking a common language which bridges genes, sequences, structures and functions ensures that we understand each other and communicate scientific findings effectively while also minimizing misunderstandings and confusion [300].

## 11. Availability and Citation

Online access to IMGT^®^ databases, tools and web resources is freely available for academics. Authors are encouraged to cite the references quoted in this article. Access to IMGT^®^ databases and tools are under CNRS licenses and contracts for companies.

## Figures and Tables

**Table 1:** IMGT-ONTOLOGY axioms, concepts, IMGT Scientific chart rules and examples of IMGT expertise data concepts.

IMGT-ONTOLOGY Axioms	IMGT-ONTOLOGY Concepts	IMGT Scientific Chart Rules	Examples of IMGT Expertise Data Concepts
IDENTIFICATION axiom [50]	Concepts of identification [50]	IMGT standardized keywords (e.g., reference sequence, clonotype, paratope, epitope, allotype, variant, Fc receptor, FcR) (1)	Molecule type, receptor type, chain type, gene type, configuration type, molecule entity type, functionality [50]
DESCRIPTION axiom [51]	Concepts of description [51]	IMGT standardized labels and annotations (e.g., V-REGION, CDR-IMGT, FR-IMGT, antibody description) IMGT prototypes	Core (V-, D-, J-, C-) Prototypes Labels for sequences Labels for 2D and 3D structures [51]
CLASSIFICATION axiom [52] ‘one of the two pillars of immunoinformatics’ [66]	Concepts of classification (group, subgroup, gene, allele) [52]	IMGT standardized IG and TR gene nomenclature (group, subgroup, gene, allele) [2,6,12,13] IMGT clans (between species)	Gene tables Alignments of alleles Tables of alleles IMGT Reference sequences directories (IG and TR for all jawed vertebrate species)
NUMEROTATION axiom [53,54,55,56,57,58,59,60,61,62,63,64,65,66] ‘one of the two pillars of immunoinformatics’ [66]	Concepts of numerotation (IMGT unique numbering, IMGT Collier de Perles) [53,54,55,56,57,58,59,60,61,62,63,64,65,66]	IMGT unique numbering for: V- and V-LIKE-DOMAIN [55] C- and C-LIKE-DOMAIN [56] G- and G-LIKE-DOMAIN [57] IMGT Colliers de Perles [61,62,63,64,65,66]	Protein displays IMGT Colliers de Perles for V, C and G domains FR-IMGT and CDR-IMGT delimitations
LOCALIZATION axiom	Concepts of localization [48]	5′ borne and 3′ borne Copy number variation (CNV)	Chromosomal localization Locus representation Standardized keywords
ORIENTATION axiom [48,49]	Concepts of orientation [48,49]	Orientation of genomic instances relative to each other (i.e., centromeric, telomeric, 5′, 3′)	Chromosome orientation Locus orientation Gene orientation DNA strand orientation Domain beta-strand orientation
OBTENTION axiom [48,49]	Standardized origin Standardized methodology [48,49]	Standardized origin Standardized methodology	References

(1) Owing to the diversity and multiplicity of the Fc gamma receptors which belong to the related proteins of immune interest (RPIs), and in the absence of standardized sequence characterization in functional analysis, these receptors are usually identified with keywords, for example for *Homo sapiens*, FcγR, FcγRI, FcγRII, FcγRIII, etc. However, it should be noted that, when there is no ambiguity as to the interactive chain involved, the HGNC gene name should be used (FCGR1A, FCGR2A, FCGR2B, FCRG2C, FCGR3A and FCGR3B). This rule is applied for the neonatal Fc receptor (FcRn), which is made of the interactive Fc gamma receptor and transporter (FCGRT) chain associated with B2M.

**Table 2:** The ten most classical IG and TR molecule entity types and associated prototypes are shown with examples of gene and allele names. Molecule entity types are identified with IMGT standardized keywords [50] (IDENTIFICATION axiom [50]). Molecule entity prototypes are described with the corresponding longest IMGT standardized label (in capital letters [51] (DESCRIPTION axiom [51])). Examples of *Homo sapiens* (Homsap) IG gene and allele names [2] illustrate the IMGT standardized nomenclature [52]. Allele names of a given gene include the gene name followed by an asterisk (*) and a number starting chronologically from 01. (CLASSIFICATION axiom [52]). F: functional, ORF: open reading frame, P: pseudogene.

IDENTIFICATION (IMGT Standardized Keywords) [50]					DESCRIPTION (IMGT Standardized Labels) [51]	CLASSIFICATION (IMGT Standardized Nomenclature) [52]
Molecule Entity Type	Molecule Type	Gene Type	Configuration Type	Functionality	Molecule Entity Prototype	Gene and Allele Name (IG Examples)
V-gene	gDNA	V	germline	F, ORF, P	V-GENE	Homsap IGHV1-2*01
D-gene	gDNA	D	germline	F, ORF, P	D-GENE	Homsap IGHD1-1*01
J-gene	gDNA	J	germline	F, ORF, P	J-GENE	Homsap IGHJ1*01, Homsap IGKJ1*01, Homsap IGLJ2*01…
C-gene	gDNA	C	undefined	F, ORF, P	C-GENE	Homsap IGHM*01, Homsap IGHD*01, Homsap IGHG1*01…
V-D-J-gene	gDNA	V, D, J	rearranged	productive or unproductive	V-D-J-GENE	Homsap IGHV1-2*01- IGHD1-1*01-IGHJ1*01
V-J-gene	gDNA	V, J	rearranged	productive or unproductive	V-J-GENE	Homsap IGKV1-5*01- IGKJ1*01
L-V-D-J-C-sequence	cDNA	V, D, J, C	rearranged	productive or unproductive	L-V-D-J-C-SEQUENCE	Homsap IGHV1-2*01- IGHD1-1*01-IGHJ1*01-IGHM*01
L-V-J-C-sequence	cDNA	V, J, C	rearranged	productive or unproductive	L-V-J-C-SEQUENCE	Homsap IGKV1-5*01- IGKJ1*01-IGKC*01
V-D-J-C-sequence (chain or isotype)	protein	V, D, J, C	rearranged	productive or unproductive	V-D-J-C-SEQUENCE (or H-MU, H-DELTA, H-GAMMA1…)	Homsap IGHV1-2*01- IGHD1-1*01- IGHJ1*01-IGHM*01
V-J-C-sequence (chain or isotype)	protein	V, J, C	rearranged	productive or unproductive	V-J-C-SEQUENCE) (L-KAPPA, L-LAMBDA2…)	Homsap IGKV1-5*01- IGKJ1*01-IGKC*01, Homsap IGLV2-8*01- IGLJ2*01-IGLC2*01

**Table 3:** Relations for sequence description (LOCALIZATION axiom) [11,12].

Relation	Reciprocal Relation
‘adjacent_at_its_5_prime_to’	‘adjacent_at_its_3_prime_to’
‘included_with_same_5_prime_in’	‘includes_with_same_5_prime’
‘included_with_same_3_prime_in’	‘includes_with_same_3_prime’
‘overlaps_at_its_3_prime_with’	‘overlaps_at_its_5_prime_with’
‘included_in’	‘includes’

**Table 4:** IMGT databases, IMGT tools and IMGT Repertoire (IG and TR) (part of the IMGT Web resources). The core IMGT references databases and tools and IMGT Repertoire sections are in bold.

	IMGT Databases	IMGT Tools	IMGT Repertoire IG and TR)
Sequences	**IMGT/LIGM-DB** [69] IMGT/MH-DB (hosted at EBI) IMGT/PRIMER-DB [70,71] IMGT/CLL-DB ^a^ [72]	**IMGT/V-QUEST** [79,80,81,82,83,84,98] IMGT/JunctionAnalysis [85,86,87,88] IMGT/Decryption (internal) [89] IMGT/Automat (internal) [90,91] **IMGT/HighV-QUEST** [78,84,92,93,94,95,98] IMGT/StatClonotype [96,97] IMGT/PhyloGene [101] IMGT/Allele-Align IMGT/DomainDisplay [1]	‘**Proteins and alleles**’ [34]: Alignments of alleles IG [2] and TR [3] Tables of alleles CDR-IMGT lengths Protein displays [2,3] Allotypes Isotypes Engineered variants
Genes	**IMGT/GENE-DB** [73]	IMGT/LIGMotif [104] IMGT/LocusView IMGT/GeneView IMGT/GeneSearch IMGT/CloneSearch IMGT/GeneInfo [102,103] IMGT/GeneFrequency	‘**Locus and Genes**’ [34]: Chromosomal localizations [2,3] Locus representations [2,3] Locus descriptions Locus in genome assembly, locus bornes, gene order, CNV Gene exon/intron organization Gene exon/intron splicing sites Gene tables, Clans Potential germline repertoires Lists of IG and TR genes, groups, loci and orphons Correspondence between Nomenclatures [2,3]
Structures	**IMGT/2Dstructure-DB** **IMGT/3Dstructure-DB** [74,75,76]	**IMGT/DomainGapAlign** [75,78,105,106] IMGT/DomainDisplay [1] IMGT/DomainSuperimpose IMGT/StructuralQuery [74] IMGT/Collier-de-Perles [107]	‘**2D and 3D structures**’ [34]: IMGT classes for amino acid physicochemical properties [108] IMGT Colliers de Perles (2D representations on one layer or two layers [61,62,63,64,65] 3D representations. IMGT Colliers de Perles reference profiles [108] FR-IMGT and CDR-IMGT lengths Strands, loops and helices lengths
Therapeutical mAb, FPIA, CPCA	IMGT/mAb-DB [77,78]	Links to IMGT/2Dstructure-DB Links to IMGT/3Dstructure-DB	Format structure representations

^a^ IMGT/CLL-DB [72] contains IG sequences of chronic lymphocytic leukemia (CLL) patients, analyzed by IMGT/V-QUEST.

**Table 5:** The IMGT-LOCUS-UNIT label and its associated qualifiers and definitions [150].

IMGT-LOCUS-UNIT Label and Associated IMGT Qualifiers		Definition
IMGT label ^a^	IMGT-LOCUS-UNIT	gDNA of an immunoglobulin (IG) or T cell receptor (TR) IMGT locus unit from chromosome genomic assembly, that starts at the 5 prime (5′) end of the most 5′ IG or TR GENE-UNIT in the IMGT-LOCUS-UNIT and ends at the 3 prime (3′) end of the most 3′ IG or TR GENE-UNIT in the locus
IMGT qualifiers ^b^	IMGT_locus_3prime_borne ^c^	Name of the gene identified as the 3 prime (3′) borne of an IMGT-LOCUS-UNIT
	IMGT_locus_3prime_gene	IMGT gene name of the most 3 prime (3′) IG or TR GENE-UNIT of an IMGT-LOCUS-UNIT
	IMGT_locus_5prime_borne ^c^	Name of the gene identified as the 5 prime (5′) borne of an IMGT-LOCUS-UNIT
	IMGT_locus_5prime_gene	IMGT gene name of the most 5 prime (5′) IG or TR GENE-UNIT of an IMGT-LOCUS-UNIT
	IMGT_locus_ID	Identifier of an IMGT-LOCUS-UNIT comprising the IMGT_locus_name and a chronological number, separated with underscores
	IMGT_locus_chromosome	Chromosome identifier (with band or section if known)
	IMGT_locus_length	Length of an IMGT-LOCUS-UNIT in base pairs (bp) in the sequence
	IMGT_locus_name	Name of an IMGT-LOCUS-UNIT that includes the genus and species Latin names and the IMGT locus type (i.e., in higher vertebrates: IGH, IGK, IGL, TRA, TRB, TRG, TRD)
	IMGT_locus_orientation	Orientation of an IMGT-LOCUS-UNIT on a chromosome, is either ‘forward (FWD)’ or ‘reverse (REV)’
	IMGT_locus_positions	NCBI chromosome sequence accession with positions of the IMGT-LOCUS-UNIT

^a^ IMGT/LIGM-DB labels: https://www.imgt.org/ligmdb/label (accessed on 14 December 2025). ^b^ IMGT/LIGM-DB qualifiers: https://www.imgt.org/ligmdb/qualifier (accessed on 14 December 2025). ^c^ IMGT Borne: https://www.imgt.org/IMGTindex/IMGTborne.php (accessed on 14 December 2025).

**Table 6:** IMGT Locus 5′ and 3′ bornes of the IG and TR loci from mammal species [150] (updated 13 February 2026).

	IMGT Locus 5′ Borne			IMGT Locus 3′ Borne		
	Gene Name		Occurrence /Nb of Species	Gene Name		Occurrence /Nb of Species
IGH	N.d. ^a^		11/11	TMEM121	Transmembrane protein 121	8/11
				N.d. ^a^		3/11
IGK	PAX8	paired box 8	11/15	RPIA	ribose 5-phosphate isomerase A	15/15
	N.d. ^a^		4/15			
IGL	TOP3B	DNA topoisomerase III	6/13	RSPH14	radial spoke head 14 homolog	9/13
	SLC5A1	solute carrier family 5 member 1	3/13	VPREB3	V-set pre-B cell surrogate light chain 3	3/13
	N.d. ^a^		4/13	N.d. ^a^		1/13
TRA/TRD	OR10G3	olfactory receptor 10G3	8/12	DAD1	defender against cell death	12/12
	N.d. ^a^		4/12			
TRB	MOXD2	monooxygenase DBH-like 2	14/15	EPHB6	EPH receptor B6	15/15
	N.d. ^a^		1/15			
TRG	AMPH	amphiphysin	13/14	STARD3NL	STARD3 N-terminal like	14/14
	N.d. ^a^		1/14			

^a^ N.d.: Not defined.

**Table 7:** *Homo sapiens* IGH locus CNV haplotypes [150].

CNV	IGHV Genes	Fct	Gene Order	A	B	C	D	E	F	G		
CNV1-5prime	IGHV2-70	F, ORF	16									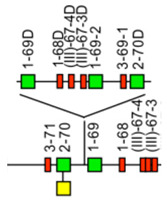
*Homo sapiens* IGH CNV1 IGHV (17–20) 7 (3F,4 P)	IGHV1-69D	F	17									
	IGHV1-68D	P	17.1									
	IGHV(III)-67-4D	P	17.2									
	IGHV(III)-67-3D	P	17.3									
	IGHV1-69-2	F	18									
	IGHV3-69-1	P	19									
	IGHV2-70D	F	20									
CNV1-3prime	IGHV1-69	F	21									
				A	B							
CNV2-5prime	IGHV4-39	F	70									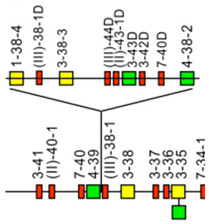
*Homo sapiens* IGH CNV2 IGHV (71–80) 10 (2F,2O,6P)	IGHV1-38-4	ORF	71									
	IGHV(III)-38-1D	P	72									
	IGHV3-38-3	ORF	73									
	IGHV(III)-44D	P	74									
	IGHV(III)-43-1D	P	75									
	IGHV3-43D	F	76									
	IGHV3-42D	P	77									
	IGHV7-40D	P	78									
	IGHV4-38-2	F	79									
	IGHV(III)-38-1	P	80									
CNV2-3prime	IGHV3-38	ORF	81									
				A	B	C	D	E	F			
CNV3-5prime	IGHV4-34	F	86									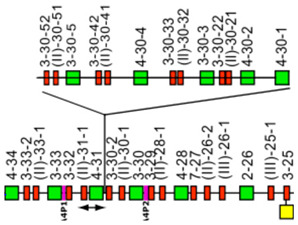
*Homo sapiens* IGH CNV3 IGHV (87–112) 26 (8F,16P,2RPI)	IGHV3-33-2	P	87									
	IGHV(II)-33-1	P	88									
	IGHV3-33	F	89									
	GOLGA4P1	nr	90									
	IGHV3-32	P	91									
	IGHV(II)-31-1	P	92									
	IGHV4-31	F	93									
	IGHV3-30-52	P	94									
	IGHV(II)-30-51	P	95									
	IGHV3-30-5	F	96									
	IGHV3-30-42	P	97									
	IGHV(II)-30-41	P	98									
	IGHV4-30-4	F	99									
	IGHV3-30-33	P	100									
	IGHV(II)-30-32	P	101									
	IGHV3-30-3	F	102									
	IGHV3-30-22	P	103									
	IGHV(II)-30-21	P	104									
	IGHV4-30-2	F	105									
	IGHV4-30-1	F	106									
	IGHV3-30-2	P	107									
	IGHV(II)-30-1	P	108									
	IGHV3-30	F	109									
	GOLGA4P2	nr	110									
	IGHV3-29	P	111									
	IGHV(II)-28-1	P	112									
CNV3-3prime	IGHV4-28	F	113									
				A	B	C	D	E	F			
CNV4-5prime	IGHV1-24	F	120									
*Homo sapiens* IGH CNV4 IGHV (121–123)3 (1F,2P)	IGHV3-23D	F	121									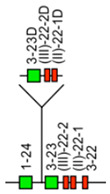
	IGHV(III)-22-2D	P	122									
	IGHV(II)-22-1D	P	123									
CNV4-3prime	IGHV3-23	F	124									
				A	B							
CNV5-5prime	IGHV3-11	F,P	144									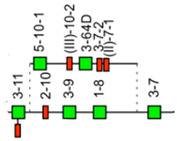
*Homo sapiens* IGH CNV5 IGHV (145–149.2) 8 (4F,4P), split into: IGHV-e1 (145–147)3 (2F,1P) IGHV-e2 (148–149.2)5 (2F,3P)	IGHV2-10	P	145									
	IGHV3-9	F	146									
	IGHV1-8	F	147									
	IGHV5-10-1	F	148									
	IGHV(III)-10-2	P	148.1									
	IGHV3-64D	F	149									
	IGHV3-7-2	P	149.1									
	IGHV(II)-7-1	P	149.2									
CNV5-3prime	IGHV3-7	F	150									
CNV6-5prime	IGHV2-5	F	154									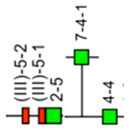
IGH CNV6	IGHV7-4-1	F	155									
IGHV (155–155.1)2(1F,1P)	IGHV(II)-4-4 *	P	155.1									
CNV6-3prime	IGHV4-4	F	156									
* IGHV(II)-4-4 is present in the CNV6 B insertion in 3′ of IGHV7-4-1.												
				A	B	C	D	E	F	G		
					I	II	III	IV	V	VI		
*Homo sapiens* IGH CNV7 IGHC (203–211) 9 (7F,1OP,1 P)	IGHG3	F	203									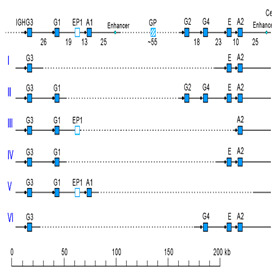
	IGHG1	F	204									
	IGHEP1	P	205									
	IGHA1	F	206									
	IGHGP	ORF, P	207									
	IGHG2	F	208									
	IGHG4	F	209									
	IGHE	F	210									
	IGHA2	F	211									
	Present in haplotype A and in other haplotype(s) of a given CNV											Absent (deletion by comparison to haplotype A of a given CNV)
	Absent in haplotype A but present by insertion in other haplotype(s) of a given CNV											Present (insertion by comparison to haplotype A of a given CNV

Genes present in haplotype A (including CNV-5prime and CNV-3prime) and in other haplotypes of a given CNV are shown in orange. A pale green color indicates the absence of a gene in haplotype A and other haplotypes, but present by insertion in other haplotype(s) of a given CNV. Genes absent, by comparison to haplotype A of a given CNV, are shown in red. Genes present, as insertion by comparison to haplotype A of a given CNV, are shown in green. Pseudogenes are written in red. F: functional, ORF: open reading frame, P: pseudogene. Letters A to G indicate the CNV haplotypes. For the CNV3, the column on the right of the haplotypes highlights the CNV3 paired motifs of the ‘duplication’, ‘triplication’ and ‘quadruplication’, with colors based on the IGHV gene functionality and subgroup or clan: green (F, IGHV4 subgroup) or blue (F, IGHV3 subgroup), dark orange (P, IGHV3 subgroup), dark red (IGHV(II) clan). The two golgin genes (GOLGA4P1 in IGHV3-33 motif and GOLGA4P2 in IGHV3-30 motif) are in yellow. An Alu sequence is present in GOLGA4P1 (M-P Lefranc, personal communication). For the CNV7, the graphical representation of the IGHC cluster from IGHG3 to IGHA2 and the multigene deletions I to VI corresponds to the haplotypes CNV7 A to G.

**Table 8:** *Homo sapiens* IG and TR IMGT locus biocuration and CNV haplotypes in genome assemblies with the example of the IGH locus (at 14q32.33). (**A**) IG and TR IMGT Locus representations and NCBI, EBI genome assemblies of the *Homo sapiens* IGH locus. (**B**) IMGT *Homo sapiens* IGH Locus CNV1-7 Haplotypes from genome assemblies.

(**A**)																																		
**IMGT** **Locus ID**					***Homo sapiens*** **IG and TR IMGT** **Locus** **Representations [2,3],** **IGH Genome** **Assemblies (NCBI, EBI)**					**Synonyms**					**Availability**					**IMGT/LIGM-DB** **IG and TR reference sequences and submission to HGNC,** **IGH GenBank** **Assembly ID**					***Homo sapiens*** **IG and TR Locus,** **Localization and Orientation on Chromosome [2,3],** **IGH locus in Genome assemblies**									
Homsap-IGH-1					The immunoglobulin FactsBooks [2], The T cell receptor FactsBook [3]										2001					IMGT/LIGM-DB IG and TR sequences annotation [2,3]. LIGM submission of 203 IG and 168 TR F and ORF (V,D,J,C) genes to HGNC [2,3].					IGH 14q32.33 (REV) [2] IGK 2p11.2 (REV) [2] IGL 22q11.2 (FWD) [2] TRA 14q11.2 (FWD) [3] TRB 7q34 (FWD) [3] TRG 7p14 (REV) [3] TRD 14q11.2 (FWD) [3]									
Homsap-IGH-2					GRCh38.p12										21 December 2017					GCA_000001405.27 GCF_000001405.38					CM000676.2 105586437106879844, complement (NC_000014.9)					IMGT000035, 1293408 bp				
Homsap-IGH-3					T2T-CHM13v2.0					hg38					24 January 2022					GCA_009914755.4 GCF_009914755.1					CP068264.2 99830032-101161492, complement					IMGT000110, 1331461 bp				
Homsap-IGH-4					GRCh38.p14					hg38					3 February 2022					GCA_000001405.29 GCF_000001405.40					CM000676.1: 106040491-107298051, complement (NC_000014.8)					IMGT000113, 1249050 bp				
F: functional, ORF: open reading frame. FWD: forward locus orientation on chromosome. REV: reverse locus orientation on chromosome.																																		
(**B**)																																		
**IMGT locus ID**							**IMGT Locus representations.** **NCBI Genomes** **assemblies**							**IMGT/LIGM-DB** **IGH locus accessionnumbers**							**IMGT/LIGM-DB IGH locus** **length (bp)**							**Homsap IGH locus** **CNV1 to CNV7**						
																												**1**	**2**	**3**	**4**	**5**	**6**	**7**
Homsap-IGH-1							FactsBook [2]							LIGM manual annotation of Homsap IGH genes locus [2]														A	A	A	A	A	A	A
Homsap-IGH-2							GRCh38.p12							IMGT000035							1293408							B	A	B	A	B	B	A
Homsap-IGH-3							T2T-CHM13v2.0							IMGT000110							1331461							A	A	C	A	A	B	H
Homsap-IGH-4							GRCh38.p14							IMGT000113							1249050							A	A	A	A	A	A	A

The letter in each column of a given row indicates the haplotype of the corresponding individual CNV (CNV1 to CNV7) for a given Homsap-IGH locus (Table 7). The 7 letters (separated by dots, in text) define the ‘IMGT Locus CNV1-7 Haplotype’ of a given Homsap-IGH locus.

**Table 9:** IMGT-NC engineered IGHG variant categories and types defined by their properties and functions [159,160].

Variant CATEGORIES	Variant Types	Property and Function Type
Effector	1	antibody-dependent cellular cytotoxicity (ADCC) reduction.
	2	antibody-dependent cellular cytotoxicity (ADCC) enhancement.
	3	antibody-dependent cellular cytotoxicity (ADCC) and antibody-dependent cellular phagocytosis (ADCP) enhancement.
	4	complement-dependent cytotoxicity (CDC) enhancement.
	5	complement-dependent cytotoxicity (CDC) reduction.
	6	antibody-dependent cellular cytotoxicity (ADCC) and complement-dependent cytotoxicity (CDC) reduction.
	7	FcγRIIB binding increase and B cell inhibition (coengagement of antigen and FcγR on the same cell).
	8	knock out CH2 84.4 glycosylation (ADCC reduction).
Half-life	9	half-life increase or decrease.
Physicochemical properties	10	abrogation of binding to Protein A, thermal stability, pI, reduced acid-induced aggregation
Structure	11	additional intrachain disulfide bridge for domain or scFv stabilization.
	12	prevention of IgG4 half-IG exchange, AA changes or insertion at the elbow of crossovers.
	13	hexamerization.
	14	enhancement of heteropairing H-H of bispecific antibodies, knobs-into-holes, charge steering, additional H-H disulfide bridge.
	15	suppression of inter H-L and/or inter H-H disulfide bridges.
	16	site-specific drug attachment, e.g., additional cysteine.
	17	enhancement of heteropairing H-L of bispecific antibodies.
	18	control of H chain expression or half-IG exchange of bispecific IgG, subclass amino acid changes.

**Table 10:** Examples of IMGT engineered IGHG variants found in therapeutical antibodies [159,160]. The different columns correspond to the items of the standardized variant characterization detailed above.

Type	Species	IMGT Engineered Variant Name	IMGT Engineered Variant Definition	IMGT Amino Acid Changes on IGHG CH Domain ^a,b^	Amino Acid Change at the Eu-IMGT Positions	IMGT Topological Motifs Identifiable in Gene and Domain, with Positions According to the IMGT Unique Numbering ^c^	1. Property and Function	2. Property and Function
**6**	**Homsap**	**6-G1v4**	**CH2** **A114**	**CH2** **P**114 > **A** (329)	P329A	IGHG1 CH2 FG 105–117 (322–332) KVSNKA..L**P**API > KVSNKA..L**A**API	**ADCC** **reduction.** Reduces FcγR binding.	**CDC** **reduction.** Reduces C1q binding.
**6**	**Homsap**	**6-G2v3**	**CH2** **A1.2,** **A1,** **S2,** **A30,** **L92,** **S115,** **S116**	CH2 **V**1.2 > **A** (235), **G**1 > **A** (237), **P**2 > **S** (238), **H**30 > **A** (268), **V**92 > **L** (309), **A**115 > **S** (330), **P**116 > **S** (331). G2sigma	V235A, G237A, P238S, H268A, V309L, A330S, P331S	IGHG2 CH2 1.6–3 (231–239) AP.P**V**AGPS > AP.P**A**A**AS**S 23–31 (261–269) CVVVDVS**H**E > CVVVDVS**A**E 89–96 (306–313) LTV**V**HQDW > LTV**L**HQDW FG 105–117 (322–332) KVSNKG..LP**AP**I > KVSNKA..LP**SS**I	**ADCC** **reduction.** Reduces FcγR binding. Undetectable ADCC and ADCP.	**CDC** **reduction.** Reduces C1q binding. Undetectable CDC.
**6**	**Homsap**	**6-G4v4**	**CH2** **A1.3,** **A1.2**	CH2 **F**1.3 > **A** (234), **L**1.2 > **A** (235) FALA	F234A L235A	IGHG4 CH2 1.6–3 (231–239) APE**FL**GGPS > APE**AA**GGPS	**ADCC** **reduction.** Reduces FcγR binding.	**CDC** **reduction.** Reduces C1q binding.
**9**	**Homsap**	**9-G1v21**	**CH2** **Y15.1,** **T16,** **E18**	CH2 **M**15.1 > **Y** (252), **S**16 > **T** (254), **T**18 > **E** (256) YTE	M252Y, S254T, T256E	IGHG1 CH2 13–18 (249–256) DTL**M**I**S**R**T** > DTL**Y**I**T**R**E**	**Half-life increase** Enhances FCGRT binding at pH 6.0.	
**12**	**Homsap**	**12-G4v5**	**hinge** **P10**	hinge **S**10 > **P** (228)	S228P	IGHG4 hinge 1–12 (216–230) ESKYGPPCP**S**CP > ESKYGPPCP**P**CP (G1-like)	Prevents in vivo and in vitro IgG4 half-IG exchange	
**8**	**Homsap**	**8-G4v36**	**CH2** **Q84.4**	CH2 **N**84.4 > **Q** (297)	N297Q	IGHG4 CH2 83–86 (292–303) REEQF**N****..**STYRVV > REEQF**Q****..**STYRVV	**ADCC** **reduction.** Reduces FcγR binding	Owing to the absence of N-glycosylation at CH2 84.4.

^a^ Engineered amino acid changes are in bold (red before the change, green after the change). When the standardized IMGT variant nomenclature is used in antibody descriptions with available amino acid sequences (e.g., WHO INN proposed and recommended lists [161,162]), positions in the antibody chains are added between parentheses instead of the Eu-IMGT positions. ^b^ Alias variant names found in the literature are written in blue. ^c^ The topological motif (highlighted in yellow) is shown before and after the AA change(s). Amino acids of the motifs at additional positions in the IMGT unique numbering for C-domain [56] (by comparison to the V-domain IMGT unique numbering [55]) are underlined, if present. The background color indicates a reduction (pink color) or an enhancement (green color) of the involved ‘Effector’ property and function. For other properties and functions, background colors refer to the category ‘Structure’ (yellow), ‘Half-life’ (pale blue color) or ‘Physicochemical’ (pale orange). (With permission from M-P. Lefranc and G. Lefranc, LIGM, Founders and Authors of IMGT^®^, the international ImMunoGeneTics information system^®^, https://www.imgt.org).

## Data Availability

No new data were created or analyzed in this study.

## Supplementary Materials

The following supporting information can be downloaded at: https://www.mdpi.com/article/10.3390/antib15020035/s1, Table S1: List of the IMGT definitions of Encyclopedia of Systems Biology. Springer, New York, NY, 2013. Table S2: List of the IUIS-NOM-IMGT-NC reports (2017–2022).

## Abbreviations

The following abbreviations are used in this manuscript:

AI	Artificial intelligence
AIR	Adaptive immune response
AR	Antigen receptor (IG and/or TR)
ARR	Antigen receptor repertoire
BcR	B cell receptor (IG with coreceptors CD79A and CD79B)
C	Constant
CDR	Complementarity determining region
CNRS	Centre National de la Recherche Scientifique
CNV	Copy number variation
CPCA	Composite protein for clinical applications
CSR	Class switch recombination
D	Diversity
DDBJ	DNA Database of Japan
EMBL	European Molecular Biology Laboratory
Fc	Fragment crystallizable
FcR	Fc receptor
FR	Framework region
FPIA	Fusion protein for immune applications
ICI	International Congress of Immunology
IG	Immunoglobulin or antibody
IgSF	Immunoglobulin superfamily
IMGT	ImMunoGeneTics
IMGT-NC	IMGT nomenclature
INN	International Nonproprietary Name
IUIS	International Union of Immunological Societies
IUIS NOM	IUIS Nomenclature committee
J	Joining
LIGM	Laboratoire d’ImmunoGénétique Moléculaire
MH	Major histocompatibility
MhSF	MH superfamily
ORF	Open reading frame
RPI	Related protein of immune interest
SHM	Somatic hypermutation
TcR	T cell receptor (TR with coreceptors CD3)
TR	T cell receptor
UM	Université de Montpellier
V	Variable
VH	Variable domain of heavy (IG chain)
VL	Variable domain of light (IG chain)
WHO	World Health Organization
