# Supplementary material for: IMGT® Nomenclature of Immunoglobulins (IG) or Antibodies and T Cell Receptors (TR): A Common Language for Immunoinformatics and Artificial Intelligence (AI)
Source: Antibodies (Basel). 2026 Apr 15;15(2):35. doi: 10.3390/antib15020035 (PMC13113880; doi:10.3390/antib15020035)
Supplement: Supplementary file 1 [file antibodies-15-00035-s001.zip › Supplementary material_Table S1_List of the IMGT definitions of Encyclopedia of Systems Biology.pdf]

Supplementary material. Table S1. List of the IMGT definitions of Encyclopedia of Systems Biology. Springer, New York, NY, 2013.

Complementarity Determining Region (CDR-IMGT)
Constant (C) domain
Constant (C) gene
Conventional gene
Diversity (D) gene
Epitope.
Framework Region (FR-IMGT)
Functional
FunctionalityType
Gene and allele nomenclature
Groove (G) Domain
IMGT Collier de Perles
IMGT® Information System
IMGT-ONTOLOGY
IMGT-ONTOLOGY, ChainType
IMGT-ONTOLOGY, CLASSIFICATION axiom
IMGT-ONTOLOGY, ConfigurationType
IMGT-ONTOLOGY, DESCRIPTION axiom
IMGT-ONTOLOGY, DomainType
IMGT-ONTOLOGY, FunctionType
IMGT-ONTOLOGY, GeneType
IMGT-ONTOLOGY, Highconcept
IMGT-ONTOLOGY, IDENTIFICATION axiom
IMGT-ONTOLOGY, Leafconcept
IMGT-ONTOLOGY, LOCALIZATION axiom
IMGT-ONTOLOGY, LocationType
IMGT-ONTOLOGY, Molecule_EntityType
IMGT-ONTOLOGY, NUMEROTATION
IMGT-ONTOLOGY, OBTENTION axiom
IMGT-ONTOLOGY, ORIENTATION axiom
IMGT-ONTOLOGY, SpecificityType
IMGT-ONTOLOGY, StructureType
IMGT-ONTOLOGY, Unproductive
IMGT unique numbering
Immunogenetics
Immunoglobulin superfamily (IgSF)
Immunoglobulin synthesis
Immunoinformatics
Information system
Joining (J) gene
Labels and relations
MH superfamily (MhSF)
MoleculeType
Open Reading Frame (ORF)
Paratope
Productive
Pseudogene
Recombination Signal (RS)

TaxonRank
Variable (V) domain
Variable (V) gene
Ontology of IMGT®
Ontology of the IMGT® Information System

Lefranc, MP. (2013). Complementarity Determining Region (CDR-IMGT). In: Dubitzky, W., Wolkenhauer, O., Cho, KH., Yokota, H. (eds) Encyclopedia of Systems Biology. Springer, New York, NY. https://doi.org/10.1007/978-1-4419-9863-7_257. pp 451-453.

Lefranc, M-P. (2013). Constant (C) Domain. In: Dubitzky, W., Wolkenhauer, O., Cho, KH., Yokota, H. (eds) Encyclopedia of Systems Biology. Springer, New York, NY. https://doi.org/10.1007/978-1-4419-9863-7_258. pp 487-489.

Lefranc, MP. (2013). Constant (C) Gene. In: Dubitzky, W., Wolkenhauer, O., Cho, KH., Yokota, H. (eds) Encyclopedia of Systems Biology. Springer, New York, NY. https://doi.org/10.1007/978-1-4419-9863-7_659. pp 489-490.

Lefranc, M-P. (2013). Conventional Gene. In: Dubitzky, W., Wolkenhauer, O., Cho, KH., Yokota, H. (eds) Encyclopedia of Systems Biology. Springer, New York, NY. https://doi.org/10.1007/978-1-4419-9863-7_660. pp 499-500.

Lefranc, M-P. (2013). Diversity (D) Gene. In: Dubitzky, W., Wolkenhauer, O., Cho, KH., Yokota, H. (eds) Encyclopedia of Systems Biology. Springer, New York, NY. https://doi.org/10.1007/978-1-4419-9863-7_661. pp 606-607.

Lefranc, MP. (2013). Epitope. In: Dubitzky, W., Wolkenhauer, O., Cho, KH., Yokota, H. (eds) Encyclopedia of Systems Biology. Springer, New York, NY. https://doi.org/10.1007/978-1-4419-9863-7_663. pp 672-673.

Lefranc, MP. (2013). Framework Region (FR-IMGT). In: Dubitzky, W., Wolkenhauer, O., Cho, KH., Yokota, H. (eds) Encyclopedia of Systems Biology. Springer, New York, NY. https://doi.org/10.1007/978-1-4419-9863-7_262. pp 758-759.

Lefranc, M-P. (2013). Functional. In: Dubitzky, W., Wolkenhauer, O., Cho, KH., Yokota, H. (eds) Encyclopedia of Systems Biology. Springer, New York, NY. https://doi.org/10.1007/978-1-4419-9863-7_664. pp 771-772.

(2013). FunctionalityType. In: Dubitzky, W., Wolkenhauer, O., Cho, KH., Yokota, H. (eds) Encyclopedia of Systems Biology. Springer, New York, NY. https://doi.org/10.1007/978-1-4419-9863-7_100529. pp 777-778.

Lefranc, M-P. (2013). Gene and Allele Nomenclature. In: Dubitzky, W., Wolkenhauer, O., Cho, KH., Yokota, H. (eds) Encyclopedia of Systems Biology. Springer, New York, NY. https://doi.org/10.1007/978-1-4419-9863-7_125. pp 787-788.

Lefranc, M-P. (2013). Groove (G) Domain. In: Dubitzky, W., Wolkenhauer, O., Cho, KH., Yokota, H. (eds) Encyclopedia of Systems Biology. Springer, New York, NY. https://doi.org/10.1007/978-1-4419-9863-7_263. pp 872-873.

Lefranc, M-P. (2013). IMGT Collier de Perles. In: Dubitzky, W., Wolkenhauer, O., Cho, KH., Yokota, H. (eds) Encyclopedia of Systems Biology. Springer, New York, NY. https://doi.org/10.1007/978-1-4419-9863-7_128. pp 944-952 (3 fig).

Lefranc, M-P. (2013). IMGT® Information System. In: Dubitzky, W., Wolkenhauer, O., Cho, KH., Yokota, H. (eds) Encyclopedia of Systems Biology. Springer, New York, NY. https://doi.org/10.1007/978-1-4419-9863-7_106. pp 959-964 (2 fig).

Giudicelli, V., Lefranc, M-P. (2013). IMGT-ONTOLOGY. In: Dubitzky, W., Wolkenhauer, O., Cho, KH., Yokota, H. (eds) Encyclopedia of Systems Biology. Springer, New York, NY. https://doi.org/10.1007/978-1-4419-9863-7_123. pp 964-972.

Lefranc, M-P. (2013). IMGT-ONTOLOGY, ChainType. In: Dubitzky, W., Wolkenhauer, O., Cho, KH., Yokota, H. (eds) Encyclopedia of Systems Biology. Springer, New York, NY. https://doi.org/10.1007/978-1-4419-9863-7_657. pp 972-973.

Lefranc, M-P. (2013). IMGT-ONTOLOGY, CLASSIFICATION Axiom. In: Dubitzky, W., Wolkenhauer, O., Cho, KH., Yokota, H. (eds) Encyclopedia of Systems Biology. Springer, New York, NY. https://doi.org/10.1007/978-1-4419-9863-7_682. pp 973-974.

Lefranc, M-P. (2013). IMGT-ONTOLOGY, ConfigurationType. In: Dubitzky, W., Wolkenhauer, O., Cho, KH., Yokota, H. (eds) Encyclopedia of Systems Biology. Springer, New York, NY. https://doi.org/10.1007/978-1-4419-9863-7_658. pp 974-975.

Lefranc, M-P. (2013). IMGT-ONTOLOGY, DESCRIPTION Axiom. In: Dubitzky, W., Wolkenhauer, O., Cho, KH., Yokota, H. (eds) Encyclopedia of Systems Biology. Springer, New York, NY. https://doi.org/10.1007/978-1-4419-9863-7_683. pp 975-976.

Lefranc, M-P. (2013). IMGT-ONTOLOGY, DomainType. In: Dubitzky, W., Wolkenhauer, O., Cho, KH., Yokota, H. (eds) Encyclopedia of Systems Biology. Springer, New York, NY. https://doi.org/10.1007/978-1-4419-9863-7_662. pp 976-977.

Lefranc, M-P. (2013). IMGT-ONTOLOGY, FunctionType. In: Dubitzky, W., Wolkenhauer, O., Cho, KH., Yokota, H. (eds) Encyclopedia of Systems Biology. Springer, New York, NY. https://doi.org/10.1007/978-1-4419-9863-7_665. pp 977-978.

Lefranc, M-P. (2013). IMGT-ONTOLOGY, GeneType. In: Dubitzky, W., Wolkenhauer, O., Cho, KH., Yokota, H. (eds) Encyclopedia of Systems Biology. Springer, New York, NY. https://doi.org/10.1007/978-1-4419-9863-7_666. pp 978-979.

Lefranc, M-P. (2013). IMGT-ONTOLOGY, Highconcept. In: Dubitzky, W., Wolkenhauer, O., Cho, KH., Yokota, H. (eds) Encyclopedia of Systems Biology. Springer, New York, NY. https://doi.org/10.1007/978-1-4419-9863-7_684. pp 979-980.

Lefranc, M-P. (2013). IMGT-ONTOLOGY, IDENTIFICATION Axiom. In: Dubitzky, W., Wolkenhauer, O., Cho, KH., Yokota, H. (eds) Encyclopedia of Systems Biology. Springer, New York, NY. https://doi.org/10.1007/978-1-4419-9863-7_685. pp 980-981.

Lefranc, M-P. (2013). IMGT-ONTOLOGY, Leafconcept. In: Dubitzky, W., Wolkenhauer, O., Cho, KH., Yokota, H. (eds) Encyclopedia of Systems Biology. Springer, New York, NY. https://doi.org/10.1007/978-1-4419-9863-7_686. p 981.

Lefranc, M-P. (2013). IMGT-ONTOLOGY, LOCALIZATION Axiom. In: Dubitzky, W., Wolkenhauer, O., Cho, KH., Yokota, H. (eds) Encyclopedia of Systems Biology. Springer, New York, NY. https://doi.org/10.1007/978-1-4419-9863-7_687. pp 981-982.

Lefranc, M-P. (2013). IMGT-ONTOLOGY, LocationType. In: Dubitzky, W., Wolkenhauer, O., Cho, KH., Yokota, H. (eds) Encyclopedia of Systems Biology. Springer, New York, NY. https://doi.org/10.1007/978-1-4419-9863-7_669. pp 982-983.

Lefranc, M-P. (2013). IMGT-ONTOLOGY, Molecule_EntityType. In: Dubitzky, W., Wolkenhauer, O., Cho, KH., Yokota, H. (eds) Encyclopedia of Systems Biology. Springer, New York, NY. https://doi.org/10.1007/978-1-4419-9863-7_670. pp 983-984.

Lefranc, M-P. (2013). IMGT-ONTOLOGY, NUMEROTATION Axiom. In: Dubitzky, W., Wolkenhauer, O., Cho, KH., Yokota, H. (eds) Encyclopedia of Systems Biology. Springer, New York, NY. https://doi.org/10.1007/978-1-4419-9863-7_688. pp 984-985.

Lefranc, M-P. (2013). IMGT-ONTOLOGY, OBTENTION Axiom. In: Dubitzky, W., Wolkenhauer, O., Cho, KH., Yokota, H. (eds) Encyclopedia of Systems Biology. Springer, New York, NY. https://doi.org/10.1007/978-1-4419-9863-7_689. pp 985-986.

Lefranc, M-P. (2013). IMGT-ONTOLOGY, ORIENTATION Axiom. In: Dubitzky, W., Wolkenhauer, O., Cho, KH., Yokota, H. (eds) Encyclopedia of Systems Biology. Springer, New York, NY. https://doi.org/10.1007/978-1-4419-9863-7_690. pp 986-987.

Lefranc, M-P. (2013). IMGT-ONTOLOGY, SpecificityType. In: Dubitzky, W., Wolkenhauer, O., Cho, KH., Yokota, H. (eds) Encyclopedia of Systems Biology. Springer, New York, NY. https://doi.org/10.1007/978-1-4419-9863-7_677. pp 987-988.

Lefranc, M-P. (2013). IMGT-ONTOLOGY, StructureType. In: Dubitzky, W., Wolkenhauer, O., Cho, KH., Yokota, H. (eds) Encyclopedia of Systems Biology. Springer, New York, NY. https://doi.org/10.1007/978-1-4419-9863-7_678. p 988.

Lefranc, M-P. (2013). IMGT-ONTOLOGY, Unproductive. In: Dubitzky, W., Wolkenhauer, O., Cho, KH., Yokota, H. (eds) Encyclopedia of Systems Biology. Springer, New York, NY. https://doi.org/10.1007/978-1-4419-9863-7_680. pp 988-989.

Lefranc, M-P. (2013). IMGT Unique Numbering. In: Dubitzky, W., Wolkenhauer, O., Cho, KH., Yokota, H. (eds) Encyclopedia of Systems Biology. Springer, New York, NY. https://doi.org/10.1007/978-1-4419-9863-7_127. Chapter pp. 952-959 (3 fig.).

Lefranc, M-P. (2013). Immunogenetics. In: Dubitzky, W., Wolkenhauer, O., Cho, KH., Yokota, H. (eds) Encyclopedia of Systems Biology. Springer, New York, NY. https://doi.org/10.1007/978-1-4419-9863-7_259. p. 998.

Lefranc, M-P. (2013). Immunoglobulin Superfamily (IgSF). In: Dubitzky, W., Wolkenhauer, O., Cho, KH., Yokota, H. (eds) Encyclopedia of Systems Biology. Springer, New York, NY. https://doi.org/10.1007/978-1-4419-9863-7_264. pp. 998-999.

Lefranc, M-P. (2013). Immunoglobulin Synthesis. In: Dubitzky, W., Wolkenhauer, O., Cho, KH., Yokota, H. (eds) Encyclopedia of Systems Biology. Springer, New York, NY. https://doi.org/10.1007/978-1-4419-9863-7_667. pp. 999-1002 (2 fig.))

Lefranc, M-P. (2013). Immunoinformatics. In: Dubitzky, W., Wolkenhauer, O., Cho, KH., Yokota, H. (eds) Encyclopedia of Systems Biology. Springer, New York, NY. https://doi.org/10.1007/978-1-4419-9863-7_265. p 1002.

Lefranc, M-P. (2013). Information System. In: Dubitzky, W., Wolkenhauer, O., Cho, KH., Yokota, H. (eds) Encyclopedia of Systems Biology. Springer, New York, NY. https://doi.org/10.1007/978-1-4419-9863-7_260. pp 1027-1028.

Lefranc, M-P. (2013). Joining (J) Gene. In: Dubitzky, W., Wolkenhauer, O., Cho, KH., Yokota, H. (eds) Encyclopedia of Systems Biology. Springer, New York, NY. https://doi.org/10.1007/978-1-4419-9863-7_668. p 1062.

Lefranc, M-P. (2013). Labels and Relations. In: Dubitzky, W., Wolkenhauer, O., Cho, KH., Yokota, H. (eds) Encyclopedia of Systems Biology. Springer, New York, NY. https://doi.org/10.1007/978-1-4419-9863-7_126. pp 1091-1092.

Lefranc, M-P. (2013). MH Superfamily (MhSF). In: Dubitzky, W., Wolkenhauer, O., Cho, KH., Yokota, H. (eds) Encyclopedia of Systems Biology. Springer, New York, NY. https://doi.org/10.1007/978-1-4419-9863-7_261. pp 1302-1303.

Lefranc, M-P. (2013). MoleculeType. In: Dubitzky, W., Wolkenhauer, O., Cho, KH., Yokota, H. (eds) Encyclopedia of Systems Biology. Springer, New York, NY. https://doi.org/10.1007/978-1-4419-9863-7_671. pp 1455-1456.

Lefranc, M-P. (2013). Open Reading Frame (ORF). In: Dubitzky, W., Wolkenhauer, O., Cho, KH., Yokota, H. (eds) Encyclopedia of Systems Biology. Springer, New York, NY. https://doi.org/10.1007/978-1-4419-9863-7_672. pp 1566-1567.

Lefranc, M-P. (2013). Paratope. In: Dubitzky, W., Wolkenhauer, O., Cho, KH., Yokota, H. (eds) Encyclopedia of Systems Biology. Springer, New York, NY. https://doi.org/10.1007/978-1-4419-9863-7_673. pp 1632-1633.

Lefranc, M-P. (2013). Productive. In: Dubitzky, W., Wolkenhauer, O., Cho, KH., Yokota, H. (eds) Encyclopedia of Systems Biology. Springer, New York, NY. https://doi.org/10.1007/978-1-4419-9863-7_674. p 1756.

Lefranc, M-P. (2013). Pseudogene. In: Dubitzky, W., Wolkenhauer, O., Cho, KH., Yokota, H. (eds) Encyclopedia of Systems Biology. Springer, New York, NY. https://doi.org/10.1007/978-1-4419-9863-7_675. pp 1800-1801.

Lefranc, M-P. (2013). Recombination Signal (RS). In: Dubitzky, W., Wolkenhauer, O., Cho, KH., Yokota, H. (eds) Encyclopedia of Systems Biology. Springer, New York, NY. https://doi.org/10.1007/978-1-4419-9863-7_676. pp 1823-1824.

Lefranc, M-P. (2013). TaxonRank. In: Dubitzky, W., Wolkenhauer, O., Cho, KH., Yokota, H. (eds) Encyclopedia of Systems Biology. Springer, New York, NY. https://doi.org/10.1007/978-1-4419-9863-7_679. pp 2145-2146.

Lefranc, M-P. (2013). Variable (V) Domain. In: Dubitzky, W., Wolkenhauer, O., Cho, KH., Yokota, H. (eds) Encyclopedia of Systems Biology. Springer, New York, NY. https://doi.org/10.1007/978-1-4419-9863-7_266. pp 2336-2337.

Lefranc, M-P. (2013). Variable (V) Gene. In: Dubitzky, W., Wolkenhauer, O., Cho, KH., Yokota, H. (eds) Encyclopedia of Systems Biology. Springer, New York, NY. https://doi.org/10.1007/978-1-4419-9863-7_681. p 2338.

(2013). Ontology of IMGT®. In: Dubitzky, W., Wolkenhauer, O., Cho, KH., Yokota, H. (eds) Encyclopedia of Systems Biology. Springer, New York, NY. https://doi.org/10.1007/978-1-4419-9863-7_101071. p 1565

(2013). Ontology of the IMGT® Information System. In: Dubitzky, W., Wolkenhauer, O., Cho, KH., Yokota, H. (eds) Encyclopedia of Systems Biology. Springer, New York, NY. https://doi.org/10.1007/978-1-4419-9863-7_101072. p 1565
