# Supplementary material for: IMGT® Nomenclature of Immunoglobulins (IG) or Antibodies and T Cell Receptors (TR): A Common Language for Immunoinformatics and Artificial Intelligence (AI)
Source: Antibodies (Basel). 2026 Apr 15;15(2):35. doi: 10.3390/antib15020035 (PMC13113880; doi:10.3390/antib15020035)
Supplement: Supplementary file 1 [file antibodies-15-00035-s001.zip › Supplementary material_Table S2_List of the IUIS-NOM-IMGT-NC_Reports_1 -30.pdf]

Supplementary material. Table S2. List of the IUIS-NOM-IMGT-NC reports.

IUIS-NOM-IMGT-NC_Reports 1 -30 (2017-2022)

1. 1_IUIS-NOM-IMGT-NC_Report_2017_1_1226_Homsap_IGHV_261217. Eight *Homo sapiens* IGHV new alleles. Scheepers, C.; Ed.: Lefranc, M.-P. ResearchGate: Berlin, Germany, 2023; available at https://www.researchgate.net/; doi: 10.13140/RG.2.2.29315.78885
2. 2_IUIS-NOM-IMGT-NC_Report_2018_1_0724_Homsap_IGHV_240718. Three *Homo sapiens* IGHV new alleles. Watson, C., Kleinstein, S., O'Connors, K., Ed.: Lefranc, M.-P.; ResearchGate: Berlin, Germany, 2023; available at https://www.researchgate.net/; doi: 10.13140/RG.2.2.15894.01602
3. 3_IUIS-NOM-IMGT-NC_Report_2018_2_0824_Homsap_IGHG3_240818. *Homo sapiens* IGHG3*20 new allele. Augusto, D., Calonga Solís, V., Ed. :Lefranc, M.-P.; ResearchGate: Berlin, Germany, 2023; available at https://www.researchgate.net/; doi: 10.13140/RG.2.2.32671.23200
4. 4_IUIS-NOM-IMGT-NC_Report_2018-3-0912_Musputfur_TRB_120918. Forty-three *Mustela putorius furo* TRB new genes and alleles. Gerritsen, B., de Boer, R.J., Ed.: Lefranc, M.-P.; ResearchGate: Berlin, Germany, 2023; available at https://www.researchgate.net/; doi: 10.13140/RG.2.2.19249.45921
5. 5_IUIS-NOM-IMGT-NC_Report_2018-4-1027_Orycun_TRA_TRD_271018. One-hundred-eight *Oryctolagus cuniculus* TRA and TRD new genes and alleles. Mondot, S., Lantz, O., Boudinot, P., Ed.: Lefranc, M.-P.; ResearchGate: Berlin, Germany, 2023; available at https://www.researchgate.net/; doi: 10.13140/RG.2.2.25960.34566
6. 6_IUIS-NOM-IMGT-NC_Report_2018-5-1113_Homsap_IGHC_131118. Twenty-seven *Homo sapiens* IGHG new (partial) alleles. Augusto, D., Calonga Sol, V., Ed.: Lefranc, M.-P.; ResearchGate: Berlin, Germany, 2023; available at https://www.researchgate.net/; doi: 10.13140/RG.2.2.12538.57285
7. 7_IUIS-NOM-IMGT-NC_Report_2019-1-0111_Felcat_IGL_110119. Ninety-seven *Felis catus* IGL new genes and alleles. Burnett, R., Avery, A., Rout, E., Ed.: Lefranc, M.-P.; ResearchGate: Berlin, Germany, 2023; available at https://www.researchgate.net/; doi: 10.13140/RG.2.2.14216.29443
8. 8_IUIS-NOM-IMGT-NC_Report_2019-2-0111_Felcat_TRG_110119. Twenty-five *Felis catus* TRG new genes and alleles. Radtanakatikanon, A., Keller, S Ed.: Lefranc, M.-P..; ResearchGate: Berlin, Germany, 2023; available at https://www.researchgate.net/; doi: 10.13140/RG.2.2.30993.51042
9. 9_IUIS-NOM-IMGT-NC_Report_2019-3-0111_Felcat_TRG_110119. Twenty-six *Felis catus* TRG new genes and alleles. Burnett R., Avery, A., Rout, E., Ed.: Lefranc, M.-P.; ResearchGate: Berlin, Germany, 2023; available at https://www.researchgate.net/; doi: 10.13140/RG.2.2.24282.62406
10. 10_IUIS-NOM-IMGT-NC_Report_2019-4-0116_Felcat_TRB_160119. Forty-six *Felis catus* TRB new genes and alleles. Radtanakatikanon, A., Keller, S., Ed.: Lefranc, M.-P.; ResearchGate: Berlin, Germany, 2023; available at https://www.researchgate.net/; doi: 10.13140/RG.2.2.10860.85125
11. 11_IUIS-NOM-IMGT-NC_Report_2019-5-0131_Salsal_IGHV_280219. Sixty-four *Salmo salar* IGHV new genes, 30 genes (in Locus A) on Chr. 6 and 34 genes (in locus B) on Chr. 3. Krasnov, A., Boudinot, P., Ed.: Lefranc, M.-P.; ResearchGate: Berlin, Germany, 2023; available at https://www.researchgate.net/; doi: 10.13140/RG.2.2.35187.81445
12. 12_IUIS-NOM-IMGT-NC_Report_2019-6-0218_Felcat_TRA_TRD_180219. One-hundred-forty-two *Felis catus* TRA and TRD new genes and alleles. Radtanakatikanon, A., Keller, S., Ed.: Lefranc, M.-P.; ResearchGate: Berlin, Germany, 2023; available at https://www.researchgate.net/; doi: 10.13140/RG.2.2.28476.92800
13. 13_IUIS-NOM-IMGT-NC_Report_2019-7-0220_Salsal_IGHV_010. Eleven *Salmo salar* IGHV new genes and alleles. Krasnov, A., Boudinot, P., Ed.: Lefranc, M.-P.; ResearchGate: Berlin, Germany, 2023; available at https://www.researchgate.net/; doi: 10.13140/RG.2.2.15055.15527
14. 14_IUIS-NOM-IMGT-NC_Report_2019-8-0314_Homsap_IGHV_140319. Eight *Homo sapiens* IGHV new alleles. Rodriguez, O., Gibson, W.S., Silver, C.A., Smith, M., Kos, J.T., Watson, C., Ed.: Lefranc, M.-P.; ResearchGate: Berlin, Germany, 2023; available at https://www.researchgate.net/; doi: 10.13140/RG.2.2.31832.37126
15. 15_IUIS-NOM-IMGT-NC_Report_2019-9-0320_Homsap_IGHG_200319. *Homo sapiens* IGHG2 (*16, *17) and IGHG4 (*05, *06, *07, *08) new (partial) alleles. Dambrun, M., Dechavanne, C., Migot-Nabias, F., Ed.: Lefranc, M.-P.; ResearchGate: Berlin, Germany, 2023; available at https://www.researchgate.net/; doi: 10.13140/RG.2.2.25121.48481
16. 16_IUIS-NOM-IMGT-NC_Report_2019-10-0408_Oncmyk_IGH_080419. One-hundred-eighty-one *Oncorhynchus mykiss* IGH new genes and alleles: 74 genes (in locus A) on chr. 13 and 107 genes (in locus

B) on chr. 12. Magadan, S., Boudinot, P., Ed.: Lefranc, M.-P.; ResearchGate: Berlin, Germany, 2023; available at https://www.researchgate.net/; doi: 10.13140/RG.2.2.11699.71200

17. 17_IUIS-NOM-IMGT-NC-Report_2019-11-0418_Homsap_IGHV_180419. Six *Homo sapiens* IGHV new alleles. Inferred allele review committee (IARC), Ed.: Lefranc, M.-P.; ResearchGate: Berlin, Germany, 2023; available at https://www.researchgate.net/; doi: 10.13140/RG.2.2.33510.09285
18. 18_IUIS-NOM-IMGT-NC_Report_2019-12-0924_Homsap_IGHV_240919. Four *Homo sapiens* IGHV new alleles. Rodriguez, O., Gibson, W.S., Silver, C.A. , Shields, K.M. , Smith, M., Watson, C. Ed.: Lefranc, M.-P. ; ResearchGate: Berlin, Germany, 2023; available at https://www.researchgate.net/; doi: 10.13140/RG.2.2.26799.20641
19. 19_IUIS-NOM-IMGT-NC_Report_2019-16-1031_Homsap_IGLV_311019. Seven *Homo sapiens* IGLV new alleles. Rodriguez, O., Tieri, D.A. Smith, S.E., Gibson, W.S., Silver,C.A., Shields, K.M., Smith, M., Watson, C., Ed.: Lefranc, M.-P.; ResearchGate: Berlin, Germany, 2023; available at https://www.researchgate.net/; doi:10.13140/RG.2.2.30154.64968
20. 20_IUIS-NOM-IMGT-NC_Report_2019-17-1118_Homsap_IGHV_211119. Three *Homo sapiens* IGHV new alleles. Inferred allele review committee (IARC), Ed.: Lefranc, M.-P.; ResearchGate: Berlin, Germany, 2023; available at https://www.researchgate.net/; doi: 10.13140/RG.2.2.16732.87688
21. 21_IUIS-NOM-IMGT-NC_Report_2019-18-1218_Homsap_IGHV_181219. Three *Homo sapiens* IGHV new alleles. Inferred allele review committee (IARC), Ed.: Lefranc, M.-P.; ResearchGate: Berlin, Germany, 2023; available at https://www.researchgate.net/; doi: 10.13140/RG.2.2.23443.76322
22. 22_IUIS-NOM-IMGT-NC_Report_2020-1-0917_Homsap_IGLV_170920. RefSeq for two known *Homo sapiens* IGLV alleles. Dorgham, A.G., Sinclair, W.,G., Rodriguez, O., Shields, K.M., Silver, C.A., Laird, M., Collins, A., Ohlin, M., Lees, W., Corcoran, M., Scheepers, C., Watson, C., Ed.: Lefranc, M.-P.; ResearchGate: Berlin, Germany, 2023; available at https://www.researchgate.net/; doi: 10.13140/RG.2.2.36826.21440
23. 23_IUIS-NOM-IMGT-NC_Report_2020-2-1008_Homsap_IGHV_081020. Four *Homo sapiens* IGHV new alleles. Dorgham, A.G., Gibson, W.S., Rodriguez, O., Watson, C., Ed.: Lefranc, M.-P.; ResearchGate: Berlin, Germany, 2023; available at https://www.researchgate.net/; doi: 10.13140/RG.2.2.30115.32803
24. 24_IUIS-NOM-IMGT-NC_Report_2020-3-1120_Homsap_IGKV_IGLV_201120. One *Homo sapiens* IGKV and two *Homo sapiens* IGLV new alleles. Collins, A., Inferred Allele Review Committee (IARC), Ed.: Lefranc, M.-P.; ResearchGate: Berlin, Germany, 2023; available at https://www.researchgate.net/; doi: 10.13140/RG.2.2.21726.72009
25. 25_IUIS-NOM-IMGT-NC_Report_2021-1-0611_Homsap_IGKV_IGLV_220621. Five *Homo sapiens* IGKV and four *Homo sapiens* IGLV new alleles. Mikocziova, I., Peres, A., Gidoni, M., Greiff, V., Yaari, G., Sollid, L.M., Ed:. Lefranc, M.-P. and Matsuda, F.; ResearchGate: Berlin, Germany, 2023; available at https://www.researchgate.net/; doi: 10.13140/RG.2.2.22565.58085
26. 26_IUIS-NOM-IMGT-NC_Report_2021-2-0820_Oncmyk_Ar_IGHV_D_J_200821. One-hundred-twenty-five *Oncorhynchus mykiss* Arlee strain IGH genes and alleles: 66 genes (in locus A) on chr. 13 and 59 genes (in locus B) on chr. 12. Magadan, S., Boudinot, P, Ed.: Lefranc, M.-P. and Matsuda, F.; ResearchGate: Berlin, Germany, 2023; available at https://www.researchgate.net/; doi: 10.13140/RG.2.2.29276.46721
27. 27_IUIS-NOM-IMGT-NC_Report_2021-3-1117_Homsap_IGHV_171121_2. Eight *Homo sapiens* IGHV new alleles. Ohlin, M., Inferred Allele Review Committee (IARC), Ed.: Lefranc, M.-P. ResearchGate: Berlin, Germany, 2023; available at https://www.researchgate.net/; doi: 10.13140/RG.2.2.34309.63206
28. 28_IUIS-NOM-IMGT-NC_Report_2022-1-0202_Homsap_IGKV_IGLV_030322. Eight *Homo sapiens* IGKV and eight *Homo sapiens* IGLV new alleles. Watson C., Silver, C.A., Gibson, W.S., Shields, K.M., Rodriguez, O., Smith M.L., Eds.: Lefranc, M.-P. and Matsuda, F.; ResearchGate: Berlin, Germany, 2023; available at https://www.researchgate.net/; doi: 10.13140/RG.2.2.30954.18880
29. 29_IUIS-NOM-IMGT-NC_Report_2022-2-0429_Salsal_TRB_040522. One-hundred-sixty-three *Salmo salar* (Atlantic salmon) T cell receptor beta (TRB) on chr. 9 (TRB1 locus, 44 genes and TRB2 locus, 29 genes) and on chr. 1 (TRB3 locus, 59 genes, TRB4 locus, 12 genes and TRB0 scaffold, 19 genes). Grimholt,U., Eds: Lefranc, M.-P. and Matsuda, F..; ResearchGate: Berlin, Germany, 2023; available at https://www.researchgate.net/; doi: 10.13140/RG.2.2.24243.30242
30. 30_IUIS-NOM-IMGT-NC_Report_2022-3-0722_Oncmyk_TRB_220722. Two-hundred-seventeen *Oncorhynchus mykiss* (rainbow trout) Arlee T cell receptor beta (TRB) loci on chr. 25 (TRB1 locus, 50 genes and TRB2 locus, 142 genes) and on chr. 19 (TRB3 locus, 25 genes). Magadan, S., Boudinot, P., Eds: Lefranc, M.-P. and Matsuda, F..; ResearchGate: Berlin, Germany, 2023; available at https://www.researchgate.net/; doi: 10.13140/RG.2.2.10821.52962
